# Direct Growth of Transparent Boron Nitride Neutron Shielding Layer for Space Window

**DOI:** 10.1002/advs.202516390

**Published:** 2026-03-03

**Authors:** Dobin Kim, Geunpil Kim, Hwijoon Jeong, Sk Mujaffar Hossain, Satadeep Bhattacharjee, Minjae Isaac Kwon, Yeunjeong Lee, Chanhee Yang, Yeong Seok Ham, Taek‐Soo Kim, Hyowon Moon, Inkyu Park, Seung‐Cheol Lee, Jinhwan Kim, Jongbum Kim, Jaehyun Park

**Affiliations:** ^1^ Extreme Materials Research Center Korea Institute of Science and Technology (KIST) Seoul Republic of Korea; ^2^ KHU‐KIST Department of Converging Science and Technology Kyung Hee University Seoul Republic of Korea; ^3^ Nanophotonics Research Center Korea Institute of Science and Technology (KIST) Seoul Republic of Korea; ^4^ School of Electrical Engineering Korea University Seoul Republic of Korea; ^5^ Department of Applied Bioengineering Graduate School of Convergence Science and Technology Seoul National University Seoul Republic of Korea; ^6^ Indo‐Korea Science and Technology Center (IKST) Bangalore India; ^7^ Department of Physics University of Seoul (UoS) Seoul Republic of Korea; ^8^ Center for Quantum Technology Korea Institute of Science and Technology (KIST) Seoul Republic of Korea; ^9^ Department of Physics Korea University Seoul Republic of Korea; ^10^ Department of Mechanical Engineering Korea Advanced Institute of Science and Technology (KAIST) Daejeon Republic of Korea; ^11^ Nanoscience and Technology KIST School University of Science and Technology Seoul Republic of Korea; ^12^ Electronic Materials Research Center Korea Institute of Science and Technology (KIST) Seoul Republic of Korea; ^13^ Hanaro Utilization Division Korea Atomic Energy Research Institute (KAERI) Daejeon Republic of Korea

**Keywords:** direct growth, space window, thermal expansion coefficient, transparent neutron shielding layer, transparent sp^2^‐sp^3^ hybridized boron nitride

## Abstract

Cubic boron nitride (*c*‐BN) and hexagonal boron nitride (*h*‐BN) are known for their transparency and high ^10^B density, which provides a large thermal‐neutron cross‐section, yet their potential for space neutron shielding has not been explored. The fabrication of transparent *c*‐BN films remains challenging, and the chemical vapor deposition growth of *h*‐BN beyond 70 nm, or with precise thickness control and high uniformity, has not been reported except by our group. Here, we present a space window design integrating an *h*‐BN‐based neutron shielding layer with advanced ceramic bulletproof layers and a γ‐ray shielding layer. By incorporating C and O into *h*‐BN, sp^2^‐sp^3^ hybridized BN (HBN) reduces the refractive index mismatch with the SiO_2_ substrate, achieving 90.9% transmission at 550 nm at 11.9 µm thickness and enabling stable, transparent growth up to 79.2 µm with minimized thermal expansion mismatch. The optically optimized HBN (B_0.39_N_0.39_C_0.06_O_0.16_) shows reduced boron content, but the enriched formation of 63.4% *c*‐BN, with its higher boron density, compensates for this loss. The resultant density is 3.01 g cm^−3^, evaluated from neutron‐shielding probability, and HBN achieves the same neutron‐shielding efficiency as *h*‐BN at 3% reduced thickness.

## Graphical abstract

Direct growth of sp^2^‐sp^3^ hybridized BN (HBN) on quartz, enabled by an *h*‐BN buffer, produces transparent and stable films up to 80 µm thick with high transmittance and strong neutron shielding, making HBN a promising candidate for space windows in long‐term lunar missions.

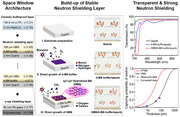

## 1 Introduction

The new space era is driving sustained human activity beyond Earth, with programs such as NASA's Artemis seeking to establish a permanent lunar foothold and prepare for Mars exploration [1, 2]. Unlike the short Apollo missions, upcoming expeditions will involve stays of ≥30–60 days, requiring infrastructure that ensures reliability, radiation protection, and operational efficiency [3]. Transparent space windows are central to this goal: they enable navigation and situational awareness while providing psychological support, yet must also withstand micrometeoroid impacts, extreme thermal swings, and ionizing radiation in the lunar environment.

A major challenge arises from low‐energy neutrons generated through regolith moderation (∼25 meV; flux ∼2 x 10^6^ cm^−2^ s^−1^), which can contribute significantly to radiation dose, induce optical degradation, and cause biological damage such as DNA alteration [4, 5, 6, 7]. Although transparent advanced bulletproof ceramics such as aluminum oxynitride (AlON) and magnesium‐aluminate spinel (MgAl_2_O_4_) provide excellent optical and mechanical properties [8, 9], no systematic strategy has integrated neutron shielding into window designs.

Low atomic number elements, such as ^6^Li and ^10^B, have been utilized as structural materials for neutron shielding due to their high 25 meV thermal‐neutron cross‐sections [10]. Among them, ^10^B offers a higher cross‐section than ^6^Li and occurs naturally at a concentration of 19.97%, making it an attractive choice for neutron shielding. Consequently, boron‐rich binary compounds, particularly cubic boron nitride (*c*‐BN) and hexagonal boron nitride (*h*‐BN), are excellent candidates for the space window. These wide‐bandgap materials are not only transparent but also exhibit excellent physical and chemical durability, as they are composed of elements with relatively low atomic numbers [11, 12].

Single‐crystal *c*‐BN can achieve ∼80% visible transmittance, limited only by reflection losses due to its high refractive index [13]. However, its synthesis requires extreme pressures (>20 GPa) and temperatures (1770–2570 K) [14], which severely restricts scalability [15]. Polycrystalline *c*‐BN exhibits ∼71% visible transmittance, limited by both reflection and absorption losses [16]. However, achieving such transmittance requires extreme synthesis conditions of ∼14 GPa pressure and ∼2000 K temperature, which hinder practical scalability.

Compared with *c*‐BN, *h*‐BN offers higher visible transmittance owing to its lower refractive index [17] and can be synthesized under relatively mild conditions, making it attractive from a manufacturing perspective. However, its practical application is limited by the mismatch in thermal expansion with transparent ceramic substrates, which induces wrinkles, cracks, and delamination in µm‐order films, as well as by its relatively low density that reduces neutron‐shielding efficiency. Reports on chemical‐vapor deposition (CVD) growth of transparent *h*‐BN beyond ∼70 nm are scarce [18], and no studies have simultaneously achieved precise thickness control and uniformity [19], apart from our previous work demonstrating the direct growth of ∼550 nm *h*‐BN on SiO_2_ with excellent thickness accuracy and homogeneity [20]. Beyond CVD, techniques such as ion‐beam‐assisted deposition, atomic‐layer deposition, and plasma‐assisted molecular beam epitaxy have been employed to grow relatively thick *h*‐BN films [21, 22, 23]; however, none have demonstrated the exceptional visible transparency of *h*‐BN due to persistent quality limitations.

Recently, density functional theory (DFT) studies have proposed a range of possible structures for sp^2^‐sp^3^ hybridized BN (HBN) [24, 25]. However, experimental demonstrations to date have been limited to plasma‐assisted laser CVD under mild conditions, producing only nanoparticles or fractal‐like morphologies [26, 27, 28]. Such forms preclude the fabrication of continuous thick films and thus prevent any meaningful assessment of their optical transparency or neutron‐shielding capability.

Here, we propose a multifunctional space‐window architecture that integrates a highly transparent HBN‐based neutron‐shielding layer into advanced ceramic bulletproof windows composed of MgAl_2_O_4_ spinel and commercial Pb‐glass for γ‐ray protection.

The HBN‐based neutron‐shielding layer was grown directly on fused quartz instead of strengthened SiO_2_ substrates, the basic repetitive units of bulletproof windows. To address the intrinsic challenges of adhesion, thermal expansion mismatch, and optical loss, we introduce a thin CVD‐grown 57 nm‐thick *h*‐BN buffer layer prior to HBN deposition. This buffer, with negligible absorption and nanoscale surface roughness, promotes controlled C and O incorporation into HBN, enhances interfacial bonding, and reduces the coefficient of thermal expansion (CTE) mismatch with quartz. As a result, continuous HBN films up to 79.2 µm in thickness were synthesized without delamination, exhibiting an adhesion energy of 1.43 J m^−2^ at 40.4 µm and full compatibility with conventional semiconductor processes [29].

The optimized HBN films display remarkable optical and shielding performance: an 11.9 µm layer achieved 90.9 % visible transmittance, only ∼2.4% lower than bare quartz, while sp^3^‐enriched HBN demonstrated equivalent thermal‐neutron attenuation with ∼3% reduced thickness compared to pure *h*‐BN. The measured density of 3.01 g cm^−3^ corresponds to a sp^3^ fraction of 63.4%, underscoring the potential of hybrid bonding to simultaneously enhance optical transparency, adhesion stability, and neutron‐shielding capacity. Together, these findings establish HBN as a viable material for transforming space windows from passive viewing elements into active radiation‐shielding components, thereby enabling safer and more sustainable human presence on the Moon and beyond.

## 2 Results and Discussion

### 2.1 Space‐Window Architecture Design for Permanent Lunar Missions and Simulation Results

**FIGURE 1:**
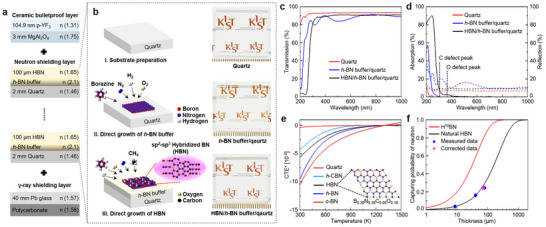
(a) Schematic illustration of the proposed space window, consisting of a MgAl_2_O_4_ ceramic bulletproof layer, a neutron shielding layer of 10 pairs of 100 µm HBN/*h*‐BN buffer/quartz, and a γ‐ray shielding layer. The p‐YF_3_ anti‐reflection coating has an optimal thickness of 104.9 nm (*n* = 1.31) for MgAl_2_O_4_. The measured *n* of *h*‐BN buffer is 2.1, and the estimated *n* of HBN is ​​1.65. The γ‐ray shielding layer is composed of a 40 mm commercial Pb glass (*n* = 1.57) coated with a polycarbonate layer (*n* = 1.58). (b) Schematics of the three‐step process for direct growth of the neutron shielding layer: I. substrate preparation, II. direct growth of *h*‐BN buffer by flowing borazine (B_3_N_6_H_6_), N_2_, O_2_, and H_2_, and III. direct growth of HBN by flowing B_3_N_3_H_6_, N_2_, CH_4_, O_2_, and H_2_. Also shown are the structural scheme of HBN and camera images of bare quartz, *h*‐BN buffer/quartz, and HBN/*h*‐BN buffer/quartz. (c) Transmission, (d) Absorption, and reflection spectra of bare quartz (red), *h*‐BN buffer/quartz (blue), and HBN/*h*‐BN buffer/quartz (black). Solid and dashed lines correspond to absorption and the reflection spectra, respectively. (e) Linear coefficients of thermal expansion relative to 1300 K (CTE*) for bare quartz, *h*‐CBN, HBN, *h*‐BN, and *c*‐BN, respectively. Inset: most stable composition of the HBN cluster. (f) Thickness‐dependent neutron‐capturing probability of H^10^BN (red) and natural HBN (black). Blue dots represent the neutron‐shielding efficiencies of 8.7, 40.4, and 79.2 µm HBNs, while the magenta star indicates corrected data for 79.2 µm HBN by considering B atomic contents.

Our space window is designed with three functional layers: an advanced ceramic bulletproof layer, a neutron shielding layer, and a γ‐ray shielding layer (**Figure** **1**a). The bulletproof layer is designed by depositing a 104.9 nm porous YF_3_ (p‐YF_3_, refractive index, *n* = 1.31 [30]) anti‐reflection coating on a MgAl_2_O_4_ ceramic substrate. Incorporation of the MgAl_2_O_4_ ceramic with repetitive strengthened‐SiO_2_ stacks enables resistance to high‐velocity projectile perforation while maintaining a reduced overall thickness [9]. Fabrication of a single HBN film with millimeter‐scale thickness is hindered by strain and rigidity at large thicknesses. As a practical strategy, we divided the total thickness into stable segments by directly growing HBN on each fused‐quartz substrate within a repetitive stack, enabling reliable thick‐film integration into the space‐window architecture.

The neutron shielding layer was directly grown on fused quartz (*n* = 1.46), instead of the conventional strengthened SiO_2_ commonly used in bulletproof windows. During the growth of *h*‐BN, control of CH_4_ and O_2_ flow resulted in HBN with a reduced CTE mismatch relative to the quartz substrate [31] and an increased *d*‐spacing that lowered the *n* [32]. Transmission spectra calculated using the transfer matrix method (Figure 1c) confirmed that the *n* of the optically optimized HBN was reduced to 1.65 (Note S1 and Figures S1 and S2).

The γ–ray shielding layer was realized by coating polycarbonate to an appropriate thickness with commercial Pb glass. For refractive index matching to minimize visible light reflection, a Pb glass (PA 90.00, SiO_2_ 68.30 wt.%, PbO 29.09 wt.%, density 2.810) [33] was selected and positioned behind the neutron shielding layer to block secondary γ–rays generated during neutron attenuation in HBN. GEANT4 water phantom simulations for neutrons and γ‐rays (Figures S3 and S4) were used to evaluate the total radiation absorbed by the human body, including secondary radiation.

The space window structure composed of 3 mm MgAl_2_O_4_, 10 pairs of 100 µm HBN/2 mm quartz, and 40 mm Pb glass (excluding polycarbonate), achieved neutron and γ‐ray shielding efficiencies of 93.9% and 99.9999%, respectively. The 10 pairs of 100 µm HBN/2 mm quartz contributed 93.6% neutron and 52.7% γ‐ray shielding, while the 40 mm Pb glass alone provided 79.4% neutron and 99.9999% γ‐ray shielding, considering secondary radiation from the multilayer structure. On the lunar surface, neutron and γ‐ray exposure levels are 63.4 and 3.4 mSv yr^−1^, respectively [4, 5]. The proposed space window yielded absorbed doses of 3.9 mSv yr^−1^ for neutrons and ∼0 mSv yr^−1^ for γ‐rays, resulting in a total of 3.9 mSv yr^−1^. As the International Commission on Radiological Protection (ICRP) recommends a maximum tolerable dose of 20 mSv yr^−1^ for human safety [34], these results confirm that the HBN‐based space window provides sufficient radiation protection while maintaining optical transparency.

### 2.2 Growth of HBN and Characterization of its Optical, Physical, and Neutron‐Shielding Properties

We directly grew the *h*‐BN buffer layer and the HBN neutron shielding layer sequentially on a quartz substrate in a non‐catalytic manner [20] without any additional transfer process, as illustrated in Figure 1b. The detailed growth setup is provided in Note S2 and Figure S5. This direct‐growth approach enabled uniform HBN growth with precise thickness control [18, 19, 20]. The *h*‐BN buffer layer was directly grown on a quartz substrate by controlling the flows of H_2_, O_2_, and borazine (B_3_N_3_H_6_) using N_2_ as carrier gas (See Methods). The *h*‐BN buffer layer was grown continuously under the same growth environment by varying the temperature profile, creating a quality gradient to optimize the interface for subsequent HBN deposition. The thickness of the *h*‐BN buffer grown on quartz was estimated by assuming that it was identical to that of *h*‐BN simultaneously grown on a SiO_2_/Si substrate under the same growth conditions. Based on the color index analysis of the *h*‐BN buffer on the SiO_2_/Si reference substrate, the thickness was estimated to be approximately 57 nm [20] (Note S3 and Figure S6). XPS analysis of the grown *h*‐BN buffer confirmed that it was composed of sp^2^‐bonded BN, with a B─O portion of ∼4.3% and an N─H portion of ∼2.9% (Note S4 and Figure S7).

For stable HBN growth, two different growth processes were tested. Detailed growth profiles of continuous and discontinuous processes are attached to Note S5 and Figure S8a,b. We chose the discontinuous growth process for HBN, which exhibited more stable and uniform elemental depth profiles with a 3.6% increase in O contents compared to the continuous process (Figure S8c,d). Structural analysis of the grown HBN revealed that it is composed of sp^2^ BN, sp^3^ BN, and anti‐stacking fault sp^2^ BN induced by sp^3^ BN formation. A schematic illustration of this structure is provided in Figure 1b. Camera images of a bare quartz substrate, an *h*‐BN buffer grown on quartz (*h*‐BN buffer/quartz), and an HBN grown on the *h*‐BN buffer/quartz (HBN/*h*‐BN buffer/quartz) are shown in Figure 1b. The *h*‐BN buffer/quartz appears brighter than bare quartz due to its higher refractive index (*n* ∼2.1). In contrast, the HBN/*h*‐BN buffer/quartz sample exhibits brightness and transparency comparable to bare quartz, indicating that the optical properties of the quartz substrate are effectively preserved after HBN growth.

Figure 1c,d shows the ultraviolet‐visible spectroscopy (UV–vis) spectra of bare quartz, *h*‐BN buffer/quartz, and optically optimized 11.9 µm HBN/*h*‐BN buffer/quartz, respectively. At 550 nm, the transmission is 93.3% for bare quartz, 84.8% for the *h*‐BN buffer/quartz, and 90.9% for the HBN/*h*‐BN buffer/quartz (Figure 1c). The reduced transmission of *h*‐BN buffer/quartz (84.8%) originates from reflection loss at the *h*‐BN/quartz interface, which induces multiple reflection interference [35], resulting in the observed oscillatory pattern in both transmission and reflection spectra (Figure 1d). After the growth of an 11.9 µm HBN layer, the visible light transmission shows only a 2.4% decrease at 550 nm compared to bare quartz without an interference pattern.

As shown in Figure 1d, the *h*‐BN buffer/quartz spectrum exhibits a peak centered at 210 nm, corresponding to the optical bandgap of *h*‐BN [36], without additional features from C [37] or O [38]. In contrast, the HBN/*h*‐BN buffer/quartz spectrum exhibits a redshifted peak centered at 244.7 nm, which can be attributed to defect‐level formation and tensile strain during the cooling process. Additionally, two more peaks are observed: one at 305 nm associated with C [37] and another at 365 nm associated with O [38]. In particular, the tailing of the O‐related peak induces an absorption loss of approximately 0.3% at 550 nm.

The HBN sample exhibits a higher reflection of 2.1% than bare quartz, resulting in a 2.4% decrease in transmission. From the transmission spectrum measured in the 400–800 nm range, the *n* of HBN was approximated to be 1.65 using the transfer matrix method (Note S1 and Figures S1 and S2).

When *h*‐BN was grown directly on a quartz substrate to micrometer‐scale thickness, cracks, wrinkles, and delamination were frequently observed. To mitigate these issues, C and O were experimentally incorporated, and the coefficient of thermal expansion (CTE) was calculated to evaluate their stabilizing effect. The composition of optically optimized HBN was determined as sp^2^ B_0.38_N_0.38_C_0.06_O_0.18_, which represents the thermodynamically most stable form predicted by cluster expansion calculations (inset of Figure 1e) [39, 40]. This composition was adopted in our work to enhance thermal compatibility with quartz.

First‐principles‐based CTE calculations, performed using density functional perturbation theory (DFPT), were conducted for this optimized quaternary (B─N─C─O) HBN system and compared with pristine *h*‐BN, *h*‐CBN, and *c*‐BN. The results, including the CTE vs. temperature (K) plot (Figure 1e), are further detailed in our companion theoretical work [41]. To validate these results, the CTEs of quartz, *h*‐CBN, *h*‐BN, and *c*‐BN were also calculated. The relative CTE (CTE* = CTE (K) – CTE (1300 K)) is plotted in Figure 1e, showing that HBN exhibits a 33.7% reduction compared to *h*‐BN, though the mismatch with quartz remains larger than that of *h*‐CBN with 50% C incorporation.

Experimentally, an 8.0 µm HBN layer grown without an *h*‐BN buffer exhibited insufficient C and O incorporation, leading to limited CTE reduction and eventual delamination from quartz (Figure S9). While delamination poses challenges for the application of HBN in space windows, it may also offer opportunities for alternative applications, such as neutron protective films for space devices.

Figure 1f shows the neutron‐shielding probability of H^10^BN and natural HBN as a function of thickness [42], simulated using Monte Carlo N‐Particle (MCNP) version 6.1 at an incident neutron energy of 4.34 meV. To validate these simulations experimentally, neutron shielding measurements were conducted at the High‐flux Advanced Neutron Application Reactor (HANARO) research reactor using a cold neutron beam with an energy of 4.34 meV incident upon natural HBN with thicknesses of 8.7, 40.4, and 79.2 µm, respectively. The neutron‐shielding performance was assessed by detecting charged particles emitted from neutron‐boron absorption reactions in boron‐doped silicon using a silicon detector, both with and without the HBN films.

The experimental results agreed well with the simulated data within experimental uncertainties, confirming the reliability of the simulation model. Using this validated framework, simulations were extended to thermal neutrons at 25 meV to evaluate the neutron‐shielding efficiency of natural HBN films at the same thicknesses. The simulations predicted efficiencies of approximately 3.7%, 16.1%, and 29.1% for films of 8.7, 40.4, and 79.2 µm, respectively. However, due to the O‐rich nature of the 79.2 µm HBN, the experimentally measured shielding efficiency was 24.3%, corresponding to an optimized HBN thickness of 64.1 µm. Correcting for the B atomic content of 36.1% yielded an O‐rich BN thickness of 71.3 µm, indicated by a star in Figure 1f. The remaining thickness discrepancy is attributed to variations in the sp^3^‐BN fraction.

While material density is commonly determined using Archimedes’ method, which utilizes volume and weight, this approach is not suitable for micrometer‐scale HBN films on quartz. Therefore, we estimated the density of HBN based on the fact that its neutron‐shielding efficiency is predominantly governed by the B atomic density. Considering that the thermal‐neutron cross‐section of ^10^B (3,855 b) [10] is roughly 2000 times higher than that of N (1.9 b), C (3.5 mb) [43], and O (0.16 mb) [44], the neutron‐shielding efficiency of HBN is predominantly governed by its B atomic density.

The neutron‐shielding probability of *h*‐BN as a function of thickness can be expressed by Equation (1) from Ref. [45]:

(1) $$P\left(T_{h-\mathrm{BN}}\right)=1-\exp\left(-\frac{T_{h-\mathrm{BN}}}{\lambda_{h-\mathrm{BN}}}\right)$$

where *T_h_*_‐BN_ is the *h*‐BN thickness, and *λ_h_*_‐BN_ (237 µm for natural *h*‐BN) is the thermal‐neutron absorption length [45].

The equivalent thickness of HBN, providing the same neutron‐shielding capability as *h*‐BN, can be calculated using Equation (2), which considers the B atomic content ratio (*α*) and the density ratio (*β*) between *h*‐BN and HBN:

(2) $$T_{\mathrm{HBN}}=\alpha \times \beta \times T_{h-\mathrm{BN}}$$

From this analysis, the equivalent thickness of HBN is 0.97 times that of *h*‐BN, indicating that HBN achieves the same shielding efficiency even at ∼3% thinner thickness (Figure S10). The corresponding absorption length of HBN, *λ*_HBN_, is 230.1 µm, obtained by multiplying *λ_h_*_‐BN_ by the equivalent thickness ratio (*α × β*). From these calculations, the density of HBN was determined to be 3.01 g cm^−3^. Further details are provided in Note S6.

### 2.3 Effect of Substrate Surface Roughness and *h*‐BN Buffer on HBN Growth

**FIGURE 2:**
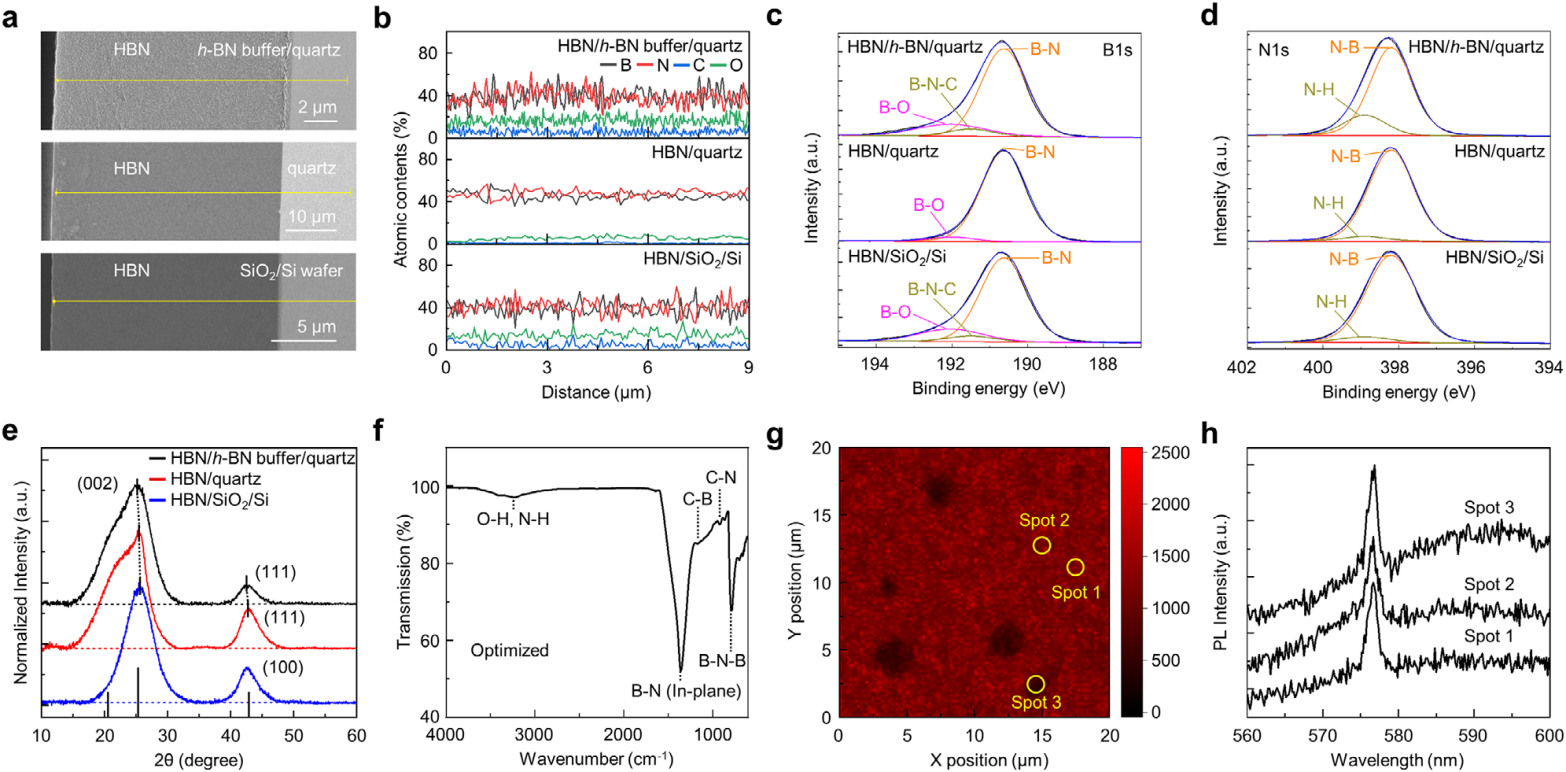
(a) Cross‐sectional SEM images of HBN/SiO_2_/Si, HBN/quartz, and HBN/*h*‐BN buffer/quartz. (b) Profiles of B, N, C, and O atomic contents for each HBN obtained along the yellow line in (a). Deconvoluted XPS spectra of (c) B1s, (d) N1s, and (e) XRD spectra of HBNs grown on each substrate. (f) FT‐IR spectrum, (g) 2D PL mapping, and (h) PL spectra corresponding to the spot marked in (g) for the optically optimized HBN/*h*‐BN buffer/quartz.

We investigated the influence of surface roughness and the presence of an *h*‐BN buffer layer on the growth of stable and thick HBN. **Figure** **2**a shows cross‐sectional scanning electron microscopy (SEM) images of HBN films grown on three types of surfaces: SiO_2_/Si wafer (the root mean square roughness (*R_q_*) of 0.3 nm), quartz (*R_q_* of 1.2 nm), and *h*‐BN buffer/quartz (*R_q_* of 6.7 nm). The AFM topography, line profiles, and corresponding *R_q_* values of the three types of substrate surfaces are provided in Figure S11. HBN films were grown directly on all substrates under optically optimized growth conditions. SEM images confirmed smooth surfaces and well‐formed interfaces for all HBN films.

Despite initially uniform growth, HBN films on SiO_2_/Si wafer and quartz without a buffer layer exhibited decreased optical transmission over time (Figure S12) and eventually delaminated from the substrate (Figure S9). In contrast, HBN films grown on the *h*‐BN buffer/quartz maintained their transmission and structural integrity over time.

Energy‐dispersive spectroscopy (EDS) line scans (Figure 2b; Figure S13) revealed compositional differences across the films. HBN/quartz without a buffer had low C and O contents (B_0.45_N_0.48_C_0.01_O_0.06_), leading to significant CTE mismatch and eventual delamination. HBN/SiO_2_/Si showed higher C and O contents (B_0.40_N_0.41_C_0.05_O_0.14_), which partially alleviated CTE mismatch, but the extremely flat surface (*R_q_* = 0.3 nm) caused insufficient adhesion and eventual delamination.

The *h*‐BN buffer layer, with a surface roughness 5.6 times larger than that of quartz and containing 4.3% O, enhanced adhesion energy with the HBN layer and facilitated the incorporation of C and O into HBN. This composition adjustment enabled better CTE matching, allowing stable growth of thick HBN films (Note S4 and Figures S7 and S11).

Figure 2c,d shows the X‐ray photoelectron spectroscopy (XPS) analysis for each HBN corresponding to Figure 2b. The B1s core‐level binding energy of *c*‐BN is typically located in the range of 190.3–190.8 eV [46, 47, 48], which is comparable to or slightly lower than the B1s peak position of *h*‐BN (190.6 eV). Similar trends are observed for the N1s spectra. In the XPS analysis of our HBN, which consists of mixed sp^2^ BN and sp^3^ BN phases, the spectra exhibited a broad and dominant B─N peak at 190.6 eV without distinct separation between sp^2^ and sp^3^ contributions. Additionally, side peaks corresponding to B─N─C (191.5 eV) and B─O (192.0 eV) were observed [49, 50]. The HBN/quartz exhibited no detectable B─N─C contribution and a B─O portion of 2.9%, whereas HBN/SiO_2_/Si showed 5.2% B─N─C and 15.7% B─O. In contrast, HBN/*h*‐BN buffer/quartz incorporated the highest levels of C and O, with 6.2% B─N─C and 16.3% B─O. Figure 2d shows the N1s spectra deconvoluted into two peaks, N─B (398.2 eV) and N─H (398.9 eV) [49]. The N─H fraction was 7.1% in HBN/quartz, 5.9% in HBN/SiO_2_/Si, and 18.5% in HBN/*h*‐BN buffer/quartz, confirming that the buffer‐assisted sample incorporated the largest proportion of N‐H.

Figure 2e shows the X‐ray diffraction (XRD) spectra from the HBNs grown on each substrate. The XRD patterns were deconvoluted into four characteristic peaks: the (002) peak at 26.7° originating from sp^2^ BN [51], the sp^2^‐sp^3^ hybridized (002) peak at 21∼22° associated with anti‐stacking faults [25, 26], the (111) peak at ∼43° from sp^3^ BN [52], and the (100) peak at ∼43° from disordered sp^2^ BN [53]. Importantly, the feature at 21∼22° is attributed solely to the sp^2^‐sp^3^ hybridized structure. HBN grown on the SiO_2_/Si wafer shows only a (002) peak at 25.5° due to the expanded d‐spacing from doping and a (100) peak originating from sp^2^ BN at 42.7°. These results indicate that the cubic phase is not induced when HBN is grown on the flat surface of the SiO_2_/Si substrate. In contrast, HBN grown on quartz displayed a shifted (002) peak at 25.4° from expanded d‐spacing, a sp^2^–sp^3^ hybridized (002) peak at 22.2° with a d‐spacing of 0.40 nm, and a (111) peak at 42.9°. Similarly, HBN grown on *h*‐BN buffer/quartz exhibited a (002) peak at 25.4° from expanded d‐spacing, a sp^2^‐sp^3^ hybridized (002) peak at 21.1° with a d‐spacing of 0.42 nm, and a sp^3^ (111) peak at 42.8°. The detailed deconvolutions of the XRD spectra are provided in Figure S14.

Figure 2f–h presents the Fourier transform infrared (FT‐IR) and photoluminescence (PL) spectra of the optically optimized HBN directly grown on the *h*‐BN buffer/quartz to further investigate its composition and structure. The FT‐IR spectrum in Figure 2f exhibits two vibrations at 786 and 1356 cm^−1^, corresponding to the sp^2^ B─N─B out‐of‐plane bending and sp^2^ B─N in‐plane stretching of *h*‐BN, respectively [54, 55]. In addition, small peaks at 920 and 1168 cm^−^^1^ are assigned to C─N and C─B bonding, respectively [56]. A weak broad peak near 3300 cm^−^^1^ is also observed, which can be attributed to overlapping N─H (3395 cm^−1^) and O─H (3231 cm^−1^) vibrations [49].

Figure 2g shows the 2D PL mapping obtained at the Raman peak positions characteristic of sp^2^ BN. The detailed PL setup is described in Note S7 and Figure S15. The mapping results indicate that HBN is predominantly synthesized in the sp^2^ BN configuration, although certain regions exhibit significantly weaker PL intensity. Figure 2h shows the PL spectra collected from three representative spots (spots 1–3) in Figure 2g, all of which exhibit emission peaks at 576 nm corresponding to the Raman feature of *h*‐BN (Figure S16) [57]. These results confirm that the synthesized HBN is overall composed of sp^2^ BN, irrespective of variations in PL intensity.

### 2.4 Changes in Optical and Physical Properties with Changes in HBN Thickness

**FIGURE 3:**
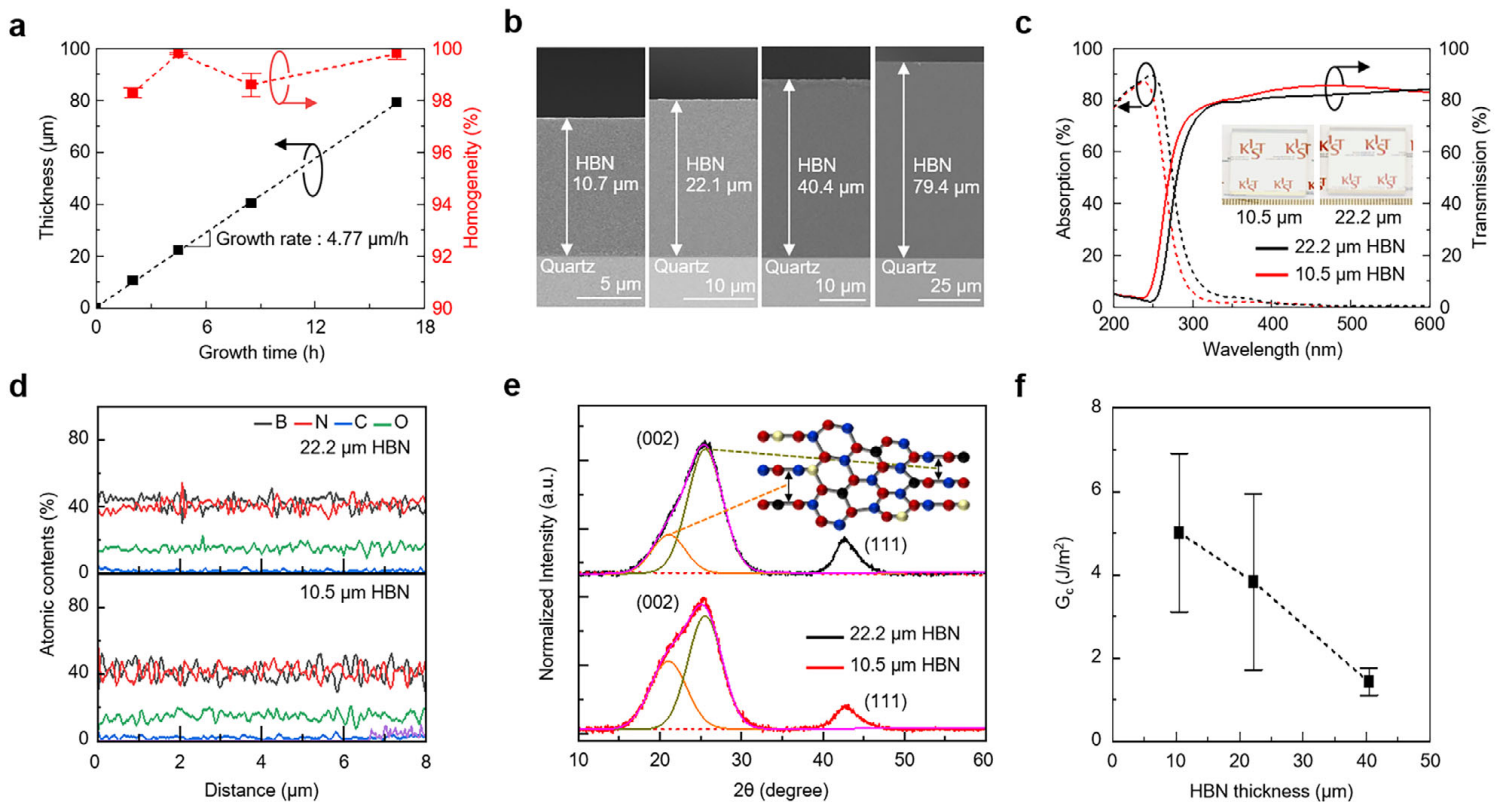
(a) Thickness and homogeneity of HBN films measured by varying the growth time. (b) Cross‐sectional SEM images of HBNs with thicknesses of 10.7, 22.1, 40.4, and 79.4 µm grown on *h*‐BN buffer/quartz. (c) Absorption (dashed) and transmission (solid) spectra of 10.5 µm (red) and 22.2 µm (black) HBNs; the inset shows camera images of each sample. (d) Profiles of B, N, C, and O atomic contents, (e) XRD spectra of 10.5 and 22.2 µm HBNs; inset shows the corresponding HBN structures for each deconvoluted (002) peak. (f) Critical adhesion energies (*G_c_*) measured by double cantilever beam (DCB) tests by varying the thicknesses of HBN.

The thickness and uniformity of HBN were measured by SEM as a function of growth time, as shown in **Figure** **3**a. A 4 cm × 4 cm HBN/quartz sample was sectioned, and cross‐sectional SEM measurements were conducted at six representative locations across the substrate. The thickness values obtained from these measurements were used to quantify the thickness uniformity in accordance with the definition and analysis procedure described in Ref. [20]. All HBNs were grown on a 57 nm *h*‐BN buffer/quartz substrate under identical gas flow conditions, and the thickness was controlled by varying the growth time. The growth rate was determined to be 4.77 µm h^−^^1^, with a thickness uniformity exceeding 98%. Figure 3b presents cross‐sectional SEM images of HBN films with thicknesses of 10.7, 22.1, 40.4, and 79.4 µm, confirming stable growth on quartz substrates.

As shown in Figure 3c, the absorption and transmission spectra of the 10.5 and 22.2 µm HBN films nearly overlap, and the inset optical camera images exhibit comparable brightness, demonstrating that film thickness has little influence on optical transparency. Only a minor increase in UV absorption (300–400 nm) is observed with increasing thickness, attributed to C and O incorporation, while absorption in the visible region remains essentially unchanged. As shown in Figure S17, the optical bandgap remains nearly constant, decreasing slightly from 4.3 to 4.2 eV, consistent with the minimal compositional variations observed in Figure 3d. The raw EDS line scan profiles of the HBN films are provided in Figure S18.

Figure 3e shows the XRD spectra of HBNs with varying thicknesses. The overall (002) peak deconvolution of both 10.5 and 22.2 µm HBN exhibited two peaks, corresponding to the (002) peak at 25.4° from sp^2^ BN and the (002) peak at 21.1° from sp^2^‐sp^3^ hybridized BN. Additionally, both 10.5 and 22.2 µm HBN showed the (111) peak at 42.7° from sp^3^ BN. As shown in the inset, the sp^2^‐sp^3^ hybridized BN structure consists of a sp^2^ BN structure with an expanded d‐spacing of 0.35 nm, a sp^2^‐sp^3^ hybridized structure with a d‐spacing of 0.42 nm [26], and a sp^3^ BN structure. As the thickness increases, the fractions of the (002) peak at 21.1° in the overall sp^2^ (002) peak decrease, and the rigidity of the HBN rises due to the increase in the (111) peak fraction, while the decrease in overall (002) peak fraction.

The sp^3^ BN fraction in HBN was estimated from the XRD spectrum of the 22.2 µm HBN film, taking into account the densities of *h*‐BN and *c*‐BN [58]. The overall (002) peak was deconvoluted into contributions from sp^2^ (002), and sp^2−^sp^3^ hybridized (002), and the density of the overall (002) structure was calculated to be 2.18 g cm^−3^, based on the atomic weight of HBN. The density of the sp^3^ (111) structure was similarly evaluated as 3.49 g cm^−3^. Since the experimentally measured density of the HBN film (Figure S10) was 3.01 g cm^−3^, the relative proportions of overall (002) and sp^3^ (111) were determined to be 36.6% and 63.4%, respectively. Accordingly, the area correction factor of the sp^3^ (111) peak relative to the overall (002) peak was calculated to be 14.69. Detailed calculation procedures are provided in Note S8 and Figure S19.

To evaluate the stability of HBN growth, the interfacial adhesion energies (*G_c_*) of HBN/*h*‐BN buffer/quartz were measured at thicknesses of 10.5, 22.2, and 40.4 µm, as shown in Figure 3f. Double cantilever beam (DCB) tests were performed, with specimens prepared using an epoxy bonding technique (Figure S20). The interfacial adhesion energy, *G_c_*, is expressed as [59]:

(3) $$G_{c}=\frac{12P_{c}^{2}a^{2}}{E^{\prime }B^{2}h^{3}}{\left(1+0.64\frac{h}{a}\right)}^{2}$$

where *E´* is the plain‐strain modulus of the fused SiO_2_, *a* is the crack length, *P_c_* is the critical load, *B* and *h* are the width and half‐height of the DCB specimen, respectively. The plane‐strain modulus (*E´*) is calculated from Hooke's law as:

(4) $$E^{\prime }=\frac{E}{1-v^{2}}$$

where *E* = 71.5 GPa [60] and *ν* = 0.17 [61] are the Young's modulus and Poisson's ratio of fused SiO_2_, giving *E*′ = 73.6 GPa. The crack length, *a*, is determined from:

(5) $$a={\left(\frac{CE^{\prime }Bh^{3}}{8}\right)}^{1/3}-0.64h$$

where *C* is the elastic compliance of the DCB specimen. During the test, repeated loading‐crack growth‐unloading cycles were performed to determine *C* and *P_c_*, which were used to calculate *G_c_*.

The measured *G_c_* decreased with increasing HBN thickness due to enhanced rigidity: 5.02 J m^−2^ at 10.5 µm, 3.84 J m^−2^ at 22.2 µm, and 1.43 J m^−2^ at 40.4 µm. These values are significantly higher than the 2.5 J m^−2^ reported for CVD‐grown monolayer *h*‐BN on Cu [62]. Despite the reduction in adhesion at increased thicknesses, the *G_c_* values are still adequate for application in standard semiconductor processing [29]. Due to the difficulty of growing a single 1 mm‐thick HBN layer with 98.5% neutron shielding in a stable form, it is essential to adopt a strategy of distributing the thickness across multiple strengthened SiO_2_ substrates for stable growth. Moreover, the stable growth of thick HBN is important not only for reducing the number of strengthened SiO_2_ substrates required but also as a key strategy to minimize optical losses at the interfaces between bonded substrates.

### 2.5 Compositional Optimization of 10 µm‐Thick HBN for High Transparency

**FIGURE 4:**
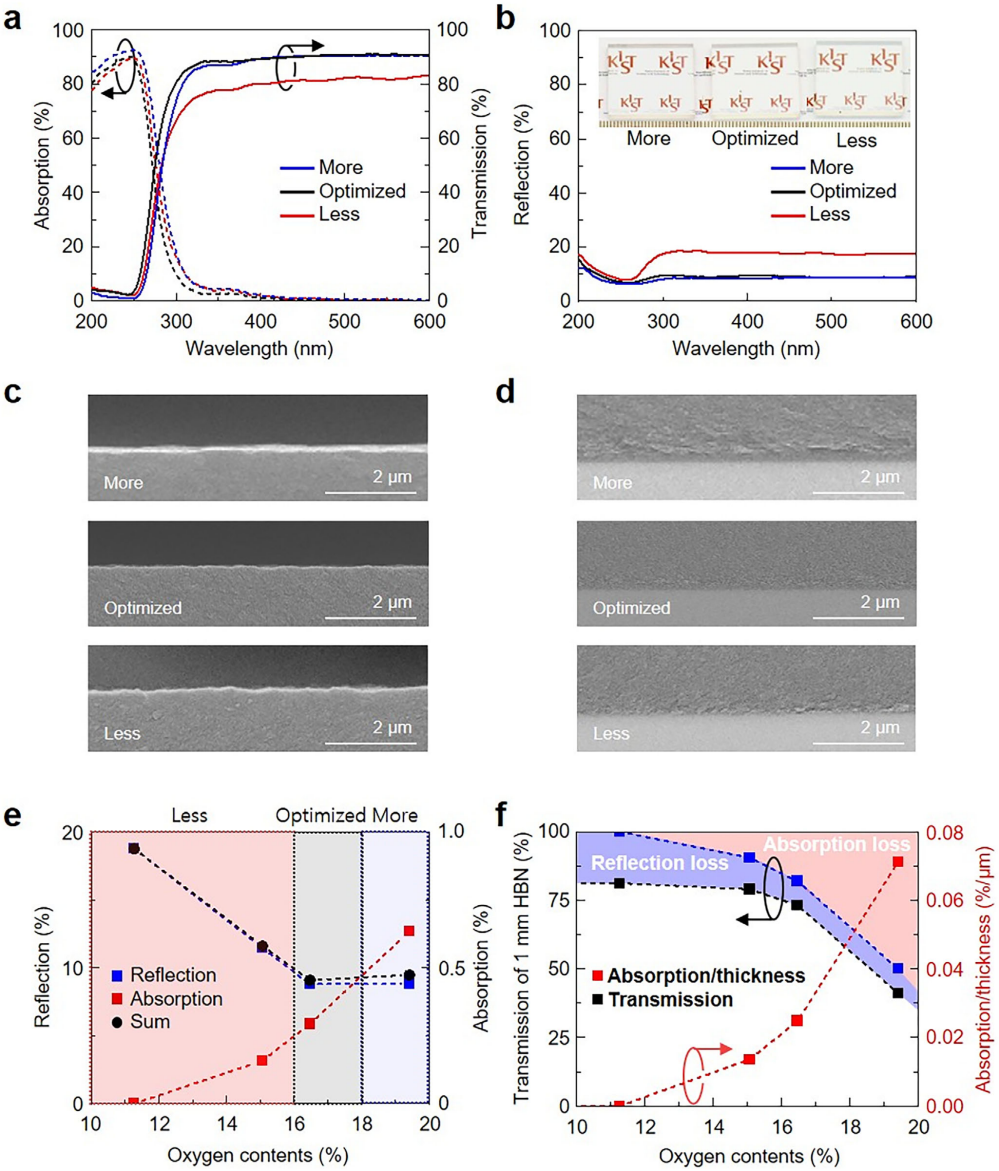
(a) Representative absorption (dashed), transmission (solid), and (b) Reflection spectra of ∼10 µm HBNs grown with varying O contents: insufficient (Less), optimized (Optimized), and excessive (More) relative to the optically optimized HBN composition. SEM images from (c) the surface and (d) the interface of each HBN. (e) Summary of the reflection (blue), absorption (red), and their sum (black) for ∼10 µm HBNs as a function of O contents. (f) Expected transmission of 1 mm HBN and absorption/thickness loss by varying O contents. The regions corresponding to absorption loss and reflection loss are sequentially indicated.

To predict the optical properties of 1 mm‐thick HBN with 98.5% neutron‐shielding efficiency, 10 µm‐thick HBN films were grown under varying stoichiometry and systematically analyzed. **Figure** **4**a,b shows the absorption, transmission, and reflection spectra of HBN categorized into three compositional groups: Less, Optimized, and More. All HBN films were grown under a constant CH_4_ flow of 5 sccm while varying the O_2_ flow from 0 to 20 sccm, resulting in approximately 5% C content. HBN with low O content is labeled as Less HBN, HBN exhibiting the highest visible light transmission and optimized O content is designated as Optimized HBN, and HBN with high O content is referred to as More HBN. Atomic compositions of these HBN films were measured by EDS (Figure S21).

Compared to Optimized HBN, More HBN shows increased UV absorption due to higher O content, although tailing in the visible region remains largely unchanged, yielding similar transmission (∼90%) in visible light. Less HBN exhibits minimal O tailing and the lowest visible absorption; however, the insufficient O content leads to higher reflection, resulting in the lowest overall transmission (Figure 4b). Camera images of More, Optimized, and Less HBN are shown in the inset of Figure 4b, where More and Optimized HBN demonstrate similar transparency, while Less HBN appears brighter due to higher reflection. This trend can be attributed to the increase in d‐spacing with higher O content in HBN, which reduces the material density and consequently lowers the refractive index. At higher O concentrations, however, the d‐spacing change becomes minimal, leading to negligible density variation and resulting in similar optical reflection.

Figure 4c,d shows the cross‐sectional SEM images showing the surface and interface of Less, Optimized, and More HBNs, respectively. No cracks or delamination were observed across the flat surfaces and interfaces, indicating that structural defects did not contribute to increased absorption or reflection, allowing for reliable optical comparisons using UV–vis measurements. Figure 4e summarizes the measured reflection and absorption as a function of O content in 10 µm HBN. As the O content increased, absorption slightly rose (<1%), whereas reflection decreased significantly from 20% to 10%. These results suggest that reducing reflection through controlled O incorporation is the dominant factor for increasing transmission in HBN films of ∼10 µm thickness. The optimum O content considering absorption was determined to be 16–18%.

Figure 4f presents the extrapolated transmission, absorption loss, and reflection loss for a 1 mm‐thick HBN with varying O contents. The reflection loss was assumed to remain constant and identical to that of the 10‐µm‐thick HBN, while the absorption loss was estimated using the absorptance derived from the Beer‐Lambert law, neglecting reflection and interference effects for simplicity. The absorptance, *A_abs_*, is expressed as:

(6) $$A_{\mathrm{abs}}=1-e^{-\mu d}$$

where *μ* is the absorption coefficient of the HBN and *d* is the film thickness. Based on this analysis, the highest predicted transmission at 1 mm thickness is 81.2%, which occurs at an O content of 11.3%. These results highlight that minimizing absorption per unit thickness is critical for realizing transparent HBN at larger thicknesses.

## 3 Conclusion

In summary, we have successfully grown highly transparent and stable HBN up to 80 µm as a neutron shielding layer on quartz substrates corresponding to strengthened SiO_2_. HBN, incorporating C and O into *h*‐BN, reduces the coefficient of thermal expansion mismatch with the growth substrate and lowers the refractive index, thereby minimizing reflection loss. The introduction of a ∼ 57 nm‐thick *h*‐BN buffer layer, with a high surface roughness of 6.7 nm and 4.3% O content, was essential for stable and transparent HBN growth by enhancing interfacial adhesion and reducing optical transmission loss.

We systematically explored HBN composition for stability and optical optimization by varying O content. The optimized composition of 11.9 µm HBN was B_0.39_N_0.39_C_0.06_O_0.16_, and the coefficient of thermal expansion simulations revealed a 33.7% reduction compared to *h*‐BN, enabling the successful growth of a 79.2 µm‐thick stable HBN. Adhesion energies (*G_c_*) up to 40.4 µm were measured and found compatible with semiconductor processes, while the neutron‐shielding efficiency was maintained even at 3% thinner than *h*‐BN. From neutron shielding‐probability analysis, the density and sp^3^ fraction of HBN were estimated to be 3.01 g cm^−3^ and 63.4%, respectively.

The optically optimized HBN exhibited 90.9% visible light transmission, confirming that reflection loss is primarily governed by differences in refractive index. Predictions for 1 mm‐thick HBNs, suitable for long‐term lunar missions, indicated that absorption loss dominates; the optimized 1 mm HBN achieved a visible light transmission of 81.2%, exceeding that of *c*‐BN (>80%).

Our results provide new insights into the design of space windows with cosmic‐radiation‐shielding capabilities for long‐term lunar missions. Considering that neutron dose levels at typical aircraft‐cruising altitudes (∼10 km) reach 51.7 mSv yr^−1^ [63, 64], corresponding to 81.5% of the lunar surface level (63.4 mSv yr^−1^) [4, 5] and far exceeding the recommended annual safety limit for aircrew (6 mSv yr^−1^), effective shielding materials such as HBN are crucial for ensuring radiation safety in both aerospace and aviation environments. Furthermore, the constituent phases of HBN, *h*‐BN, and *c*‐BN possess high thermal conductivities (408 and ∼1300 W mK^−1^, respectively) [65, 66], positioning HBN as a transparent dielectric with excellent thermal management properties. The successful demonstration of HBN integration on glass substrates highlights its potential as a high‐thermal‐conductivity transparent dielectric for next‐generation glass‐core packaging technologies.

## 4 Methods

### 4.1 Growth *h*‐BN Buffer on Quartz

For *h*‐BN buffer growth, a quartz substrate was loaded into a CVD quartz tube and heated to 900°C within 15 min under H_2_ flow (1000 sccm) at 3.4 torr. The buffer layer was deposited at 900°C for 20 min using H_2_ (1000 sccm) and borazine carried by N_2_ (100 sccm) at 3.8 torr. Subsequently, the reactor temperature was raised to 1050°C over 30 min under continuous gas flow. Finally, the N_2_ flow was stopped, and the sample was quenched to room temperature.

### 4.2 Growth of HBN on *h*‐BN Buffer/Quartz

The *h*‐BN buffer/SiO_2_ substrate was loaded into the CVD reactor and heated to 1050°C within 15 min under H_2_ flow (1000 sccm) at 3.4 torr. HBN growth was carried out at 1050°C using O_2_ (10 sccm), CH_4_ (5 sccm), and borazine delivered by N_2_ (1000 sccm) at 5.4 torr for the duration required to achieve the desired thickness. After growth, the N_2_, O_2_, and CH_4_ flows were terminated, and the sample was quenched to room temperature.

### 4.3 Characterization

UV–vis spectra were obtained using a UV–vIS–NIR spectrometer (SHIMADZU, UV‐3600) equipped with an external 3D detector (MPC‐603). SEM imaging was performed on a Regulus 8230 (Hitachi) operated at an acceleration voltage of 10 kV. The atomic contents and line scans of HBN were analyzed using Regulus8230 with an integrated EDS detector (Oxford, Extreme). XPS measurements were performed on a Nexsa (ThermoFisherScientific) at 2.0 × 10^−8^ mbar base pressure with a monochromated Al Kα (1486.6 eV) source operated at 72 W and 12 kV with a spot size of 400 × 400 µm^2^. XRD 2θ scans of HBN were obtained using a D/max‐2500/PC diffractometer. FT‐IR spectra were obtained on an iS10 spectrometer (ThermoFisher) equipped with a Ge‐ATR accessory and a DTGS detector. AFM images were acquired in contact mode using an XE‐100 microscope (Park Systems) equipped with a PPP‐CONTSCR 10M probe (Nanosensors).

## Conflicts of Interest

The authors declare no conflicts of interest.

## Supporting information




**Supporting file**: advs73855‐sup‐0001‐SuppMat.docx.

## Data Availability

The data that support the findings of this study are available from the corresponding author upon reasonable request.
