# Supplementary material for: Direct Growth of Transparent Boron Nitride Neutron Shielding Layer for Space Window
Source: Adv Sci (Weinh). 2026 Mar 3;13(20):e16390. doi: 10.1002/advs.202516390 (PMC13067756; doi:10.1002/advs.202516390)
Supplement: Supplementary file 1 — Supporting file: advs73855‐sup‐0001‐SuppMat.docx. [file ADVS-13-e16390-s001.docx]

Supporting Information

Direct Growth of Transparent Boron Nitride Neutron Shielding Layer for Space Window

Dobin Kim, Geunpil Kim, Hwijoon Jeong, Sk Mujaffar Hossain, Satadeep Bhattacharjee, Minjae Isaac Kwon, Yeunjeong Lee, Chanhee Yang, Yeong Seok Ham, Taek-Soo Kim, Hyowon Moon, Inkyu Park, Seung-Cheol Lee, Jinhwan Kim, Jongbum Kim, and Jaehyun Park*

D. Kim, J. Park

Extreme Materials Research Center, Korea Institute of Science and Technology (KIST), Seoul 02792, Republic of Korea
E-mail: jpark@kist.re.kr

D. Kim

KHU-KIST Department of Converging Science and Technology, Kyung Hee University, Seoul 02447, Republic of Korea

G. Kim, J. Kim

Nanophotonics Research Center, Korea Institute of Science and Technology (KIST), Seoul 02792, Republic of Korea

G. Kim

School of Electrical Engineering, Korea University, Seoul, 02841, Republic of Korea

J. Kim

Hanaro Utilization Division, Korea Atomic Energy Research Institute (KAERI), Daejeon 34057, Republic of Korea

H. Jeong

Department of Applied Bioengineering, Graduate School of Convergence Science and Technology, Seoul National University, Seoul, 08826, Republic of Korea

S. M. Hossain, S. Bhattacharjee, S. Lee

Indo-Korea Science and Technology Center (IKST), Bangalore-560065, India

S. Lee

Electronic Materials Research Center, Korea Institute of Science and Technology (KIST), Seoul 02792, Republic of Korea

M. I. Kwon, I. Park

Department of Physics, University of Seoul (UoS), Seoul 02504, Republic of Korea

Y. Lee, H. Moon

Center for Quantum Technology, Korea Institute of Science and Technology (KIST), Seoul 02792, Republic of Korea

Y. Lee

Department of Physics, Korea University, Seoul 02841, Republic of Korea

H. Moon

Nanoscience and Technology, KIST School, University of Science and Technology, Seoul 02792, Republic of Korea

C. Yang, Y. Ham, T. -S. Kim

Department of Mechanical Engineering, Korea Advanced Institute of Science and Technology (KAIST), Daejeon, 34141, Republic of Korea

**Supplementary Note 1. Transfer Matrix Method**

For a multilayer system of materials, the transfer matrix method is essential for simulating transmission, reflectivity, and absorption. Consider a plane wave with wavelength λ incident perpendicularly on a stack of dielectric materials with various thicknesses $t_{j}$ and refractive indices $n_{j}$. The incident light will naturally have both reflected and transmitted components, as depicted in Fig. 1. The field components for light traveling through stratified media can be solved using the transfer matrix equation $E^{'}=M_{P}M_{T}\cdot E$, where $M_{P}$ and $M_{T}$ represents the propagation matrix and transmission matrix, respectively.

To compute the propagation matrix $M_{P}$, the electric field is expressed as the sum of forward and backward propagating waves. The characteristic matrix for the $j^{th}$ layer is:

$\left( \begin{aligned} E_{+}^{'} \\ E_{-}^{'} \end{aligned} \right)=\left( \begin{matrix} e^{ik_{j}t_{j}} & 0 \\ 0 & e^{-ik_{j}t_{j}} \end{matrix} \right)\left( \begin{aligned} E_{+} \\ E_{-} \end{aligned} \right)$.

where $k_{j}$ is the wavenumber of the $j^{th}$layer. The propagation matrix across a layer can be described as:

$M_{P}= \left( \begin{matrix} e^{ik_{j}t_{j}} & 0 \\ 0 & e^{-ik_{j}t_{j}} \end{matrix} \right)$.

At an interface, the electric field is influenced by transmission and reflection from both sides. The transfer matrix method simplifies the complex vectors of multilayer systems into Fresnel coefficients for interfaces. For normal incidence, these Fresnel equations reduce to simpler forms, making the analysis of the electric field behavior more straightforward.

$$E_{+}^{2}=\frac{2n_{1}}{n_{2}+n_{1}}{E'}_{+}^{1}+\frac{n_{2}-n_{1}}{n_{1}+n_{2}}E_{-}^{2},$$

$${E'}_{+}^{1}=\frac{n_{2}-n_{1}}{n_{2}+n_{1}}{E'}_{+}^{1}+\frac{2n_{2}}{n_{1}+n_{2}}E_{-}^{2}.$$

We can simplify this equation above in matrix form:

$$\left( \begin{aligned} E_{+}^{2} \\ E_{-}^{2} \end{aligned} \right)=\frac{1}{2}\left( \begin{matrix} {n_{1}}/{n_{2}}+1 & -({n_{1}}/{n_{2}}-1) \\ -({n_{1}}/{n_{2}}-1) & {n_{1}}/{n_{2}}+1 \end{matrix} \right)\left( \begin{aligned} {E'}_{+}^{1} \\ {E^{'}}_{-}^{1} \end{aligned} \right).$$

Hence, the transmission matrix can be written as:

$M_{T}= \frac{1}{2}\left( \begin{matrix} {n_{1}}/{n_{2}}+1 & -({n_{1}}/{n_{2}}-1) \\ -({n_{1}}/{n_{2}}-1) & {n_{1}}/{n_{2}}+1 \end{matrix} \right)$.

The total characteristic matrix, $M$, that describes all N distinct layers can then be calculated can be obtained as the product of all the individual matrices across the layers and interfaces.

$$M=\prod_{j=1}^{N} M_{j}=\left( \begin{matrix} m_{11} & m_{12} \\ m_{21} & m_{22} \end{matrix} \right).$$

When light interacts with a multilayer material stack, incident and reflected light are present on one side, and transmitted light on the other. This interaction is expressed by the following matrix equation.

$$\left( \begin{aligned} E_{trans} \\ 0 \end{aligned} \right)=\left( \begin{matrix} m_{11} & m_{12} \\ m_{21} & m_{22} \end{matrix} \right)\left( \begin{aligned} E_{inc} \\ E_{ref} \end{aligned} \right).$$

Then, the transmitted and reflected electric fields are given by

$E_{trans}= \frac{det(M)}{m_{22}}E_{inc}$ , $E_{ref}=-\frac{m_{21}}{m_{22}}E_{inc}$.

$$M=\prod_{j=1}^{N} M_{j}=\left( \begin{matrix} m_{11} & m_{12} \\ m_{21} & m_{22} \end{matrix} \right).$$

5
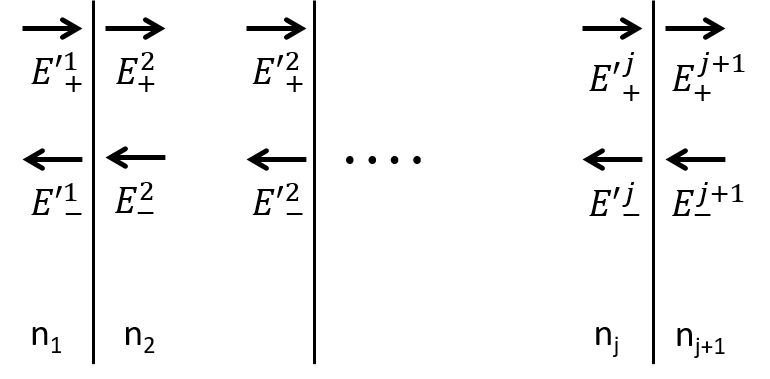


**Figure S1.** Notation of electric field amplitudes within an arbitrary multilayer.


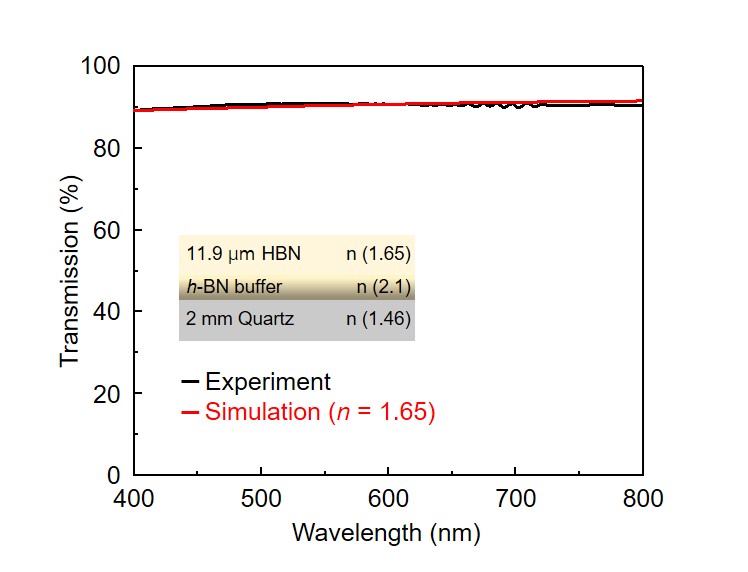


**Figure S2.** Simulation result of the *n* of sp^2^-sp^3^ hybridized BN (HBN) using the transfer matrix method. The inset shows the structure, thickness, and *n* of the simulated sample.


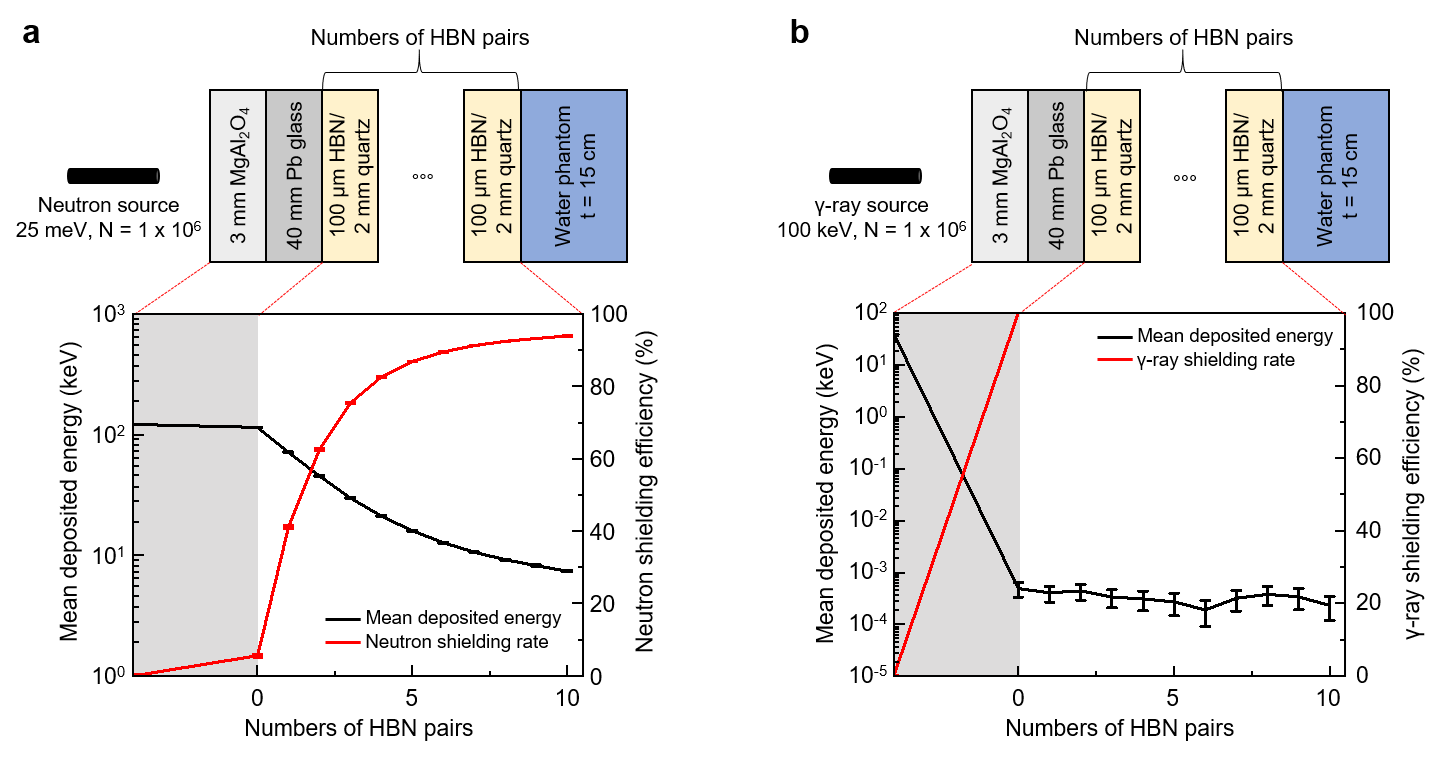


**Figure S3.** GEANT4 simulations of mean deposited energy and shielding efficiency of the space window without a polycarbonate layer using a) a 25 meV neutron source (N = 1 × 10^6^) and b) a 100 keV γ-ray source (N = 1 × 10^6^), estimated in a 15 cm water phantom. Deposited energies and shielding efficiencies of neutrons and γ-rays were calculated as a function of the number of 100 μm HBN/2 mm quartz pairs.


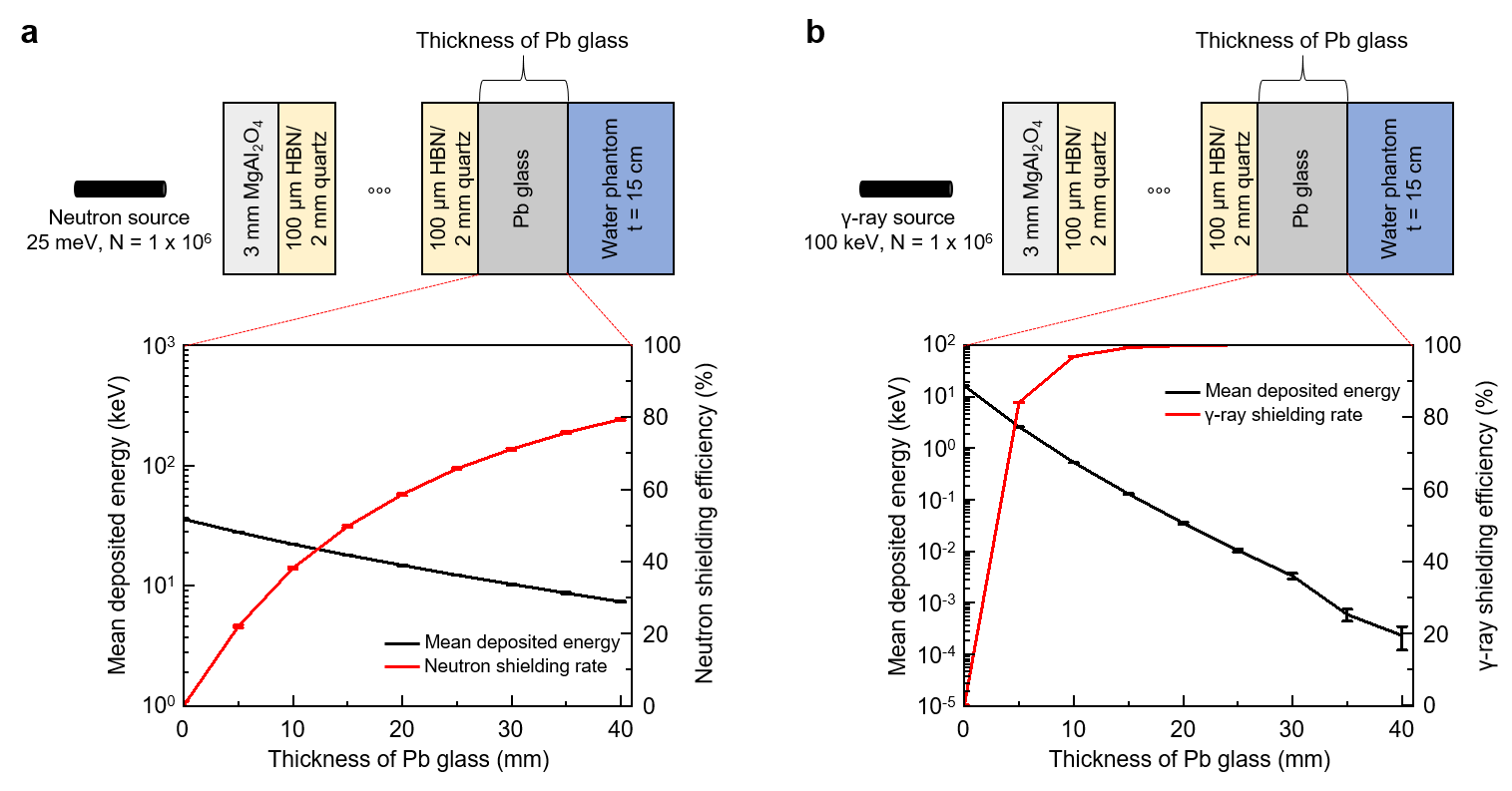


**Figure S4.** GEANT4 simulations of mean deposited energy and shielding efficiency of the space window without a polycarbonate layer using a) a 25 meV neutron source (N = 1 × 10^6^) and b) a 100 keV γ-ray source (N = 1 × 10^6^), estimated in a 15 cm water phantom. Deposited energies and shielding efficiencies of neutrons and γ-rays were calculated as a function of Pb glass thickness in 5 mm increments.

**Supplementary Note 2. Details of CVD Setup**

The chemical vapor deposition (CVD) system simply consists of a 2” quartz tube inside a high-performance SiC furnace, four mass flow controllers (MFCs), and an oil-free mechanical pump. High-purity N_2_ (99.9999%) was employed as a carrier gas to supply the vaporized borazine (B_3_N_3_H_6_, JSI Silicone Co., ≥99.9%), stored in a canister kept at -10°C to suppress self-decomposition and to control the vapor composition. The flow rate of each H_2_ (99.9999%), O_2_ (99.9992%), CH_4_ (99.95%), and N_2_ carrying B_3_N_3_H_6_ was precisely controlled by MFCs.


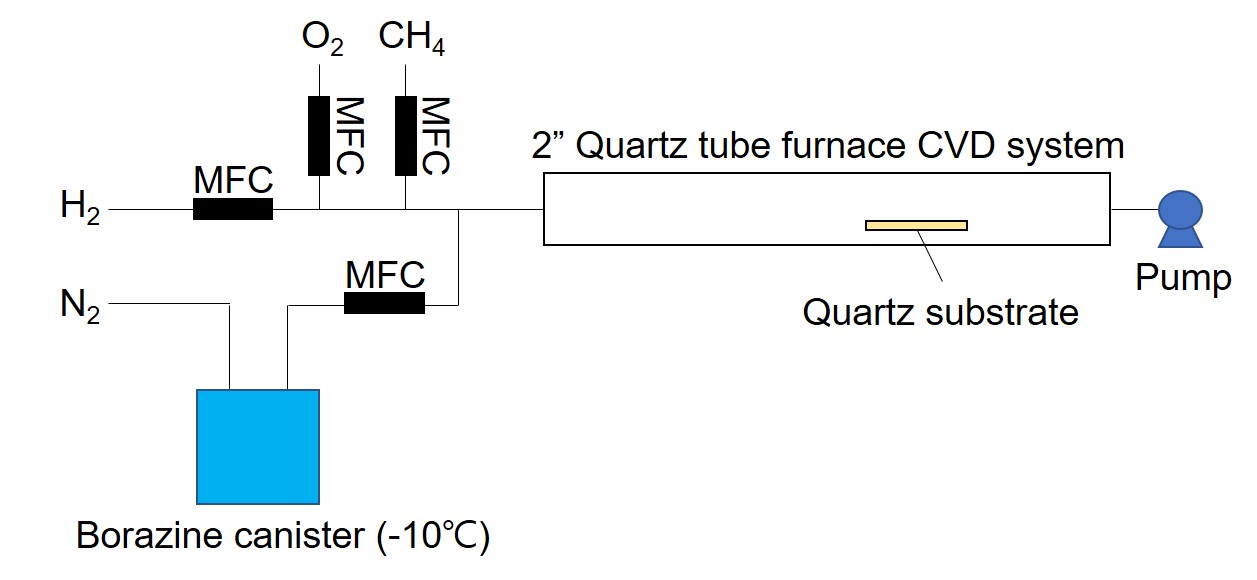


**Figure S5.** Schematic of a 2” quartz tube furnace CVD system for the growth of HBN.

**Supplementary Note 3. Thickness Estimation of *h*-BN Buffer Using the Color Index**

To estimate the thickness of the *h*-BN buffer grown on quartz, an As-doped 300 nm SiO_2_/Si wafer was simultaneously subjected to the *h*-BN buffer growth and used as a thickness reference sample. The thickness of the *h*-BN buffer grown on quartz was assumed to be identical to that of the reference sample. The thickness was determined by comparing the *h*-BN color index samples with known thicknesses previously measured by atomic force microscopy (AFM) ^[1]^. A perpendicular line was drawn from the selected index point to the guideline, and the thickness was estimated to be ~57 nm, based on the distance ratio between the reference thicknesses of 51.7 nm and 70.5 nm.


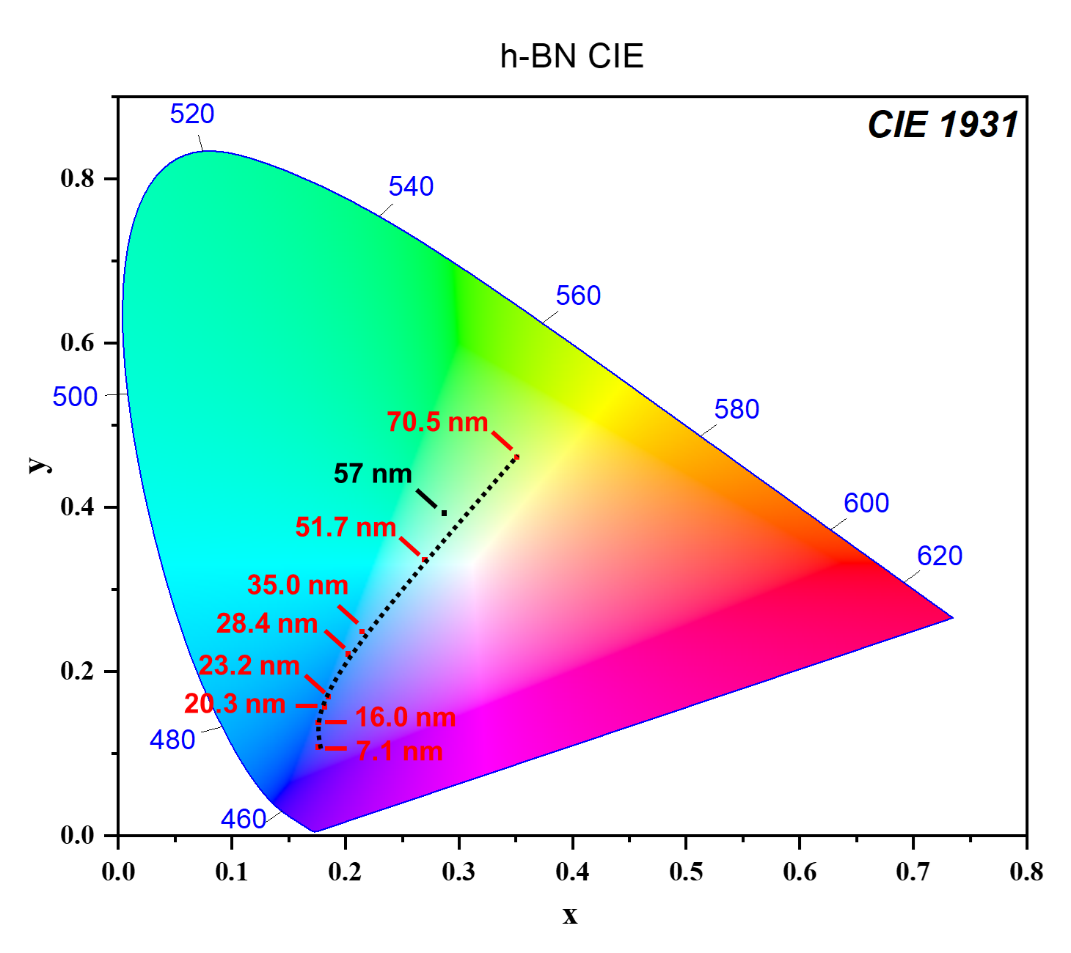


**Figure S6.** Color index plot of the *h*-BN index samples (red dot) and the *h*-BN buffer reference sample (black dot).

**Supplementary Note 4. Deconvolutions of XPS Spectra for *h*-BN buffer/Quartz Sample**

The B1s spectrum was deconvoluted into two peaks centered at 190.7 eV and 192.0 eV, corresponding to B–N and B–O bonds, respectively, with the B–O portion estimated to be 4.3%. The N1s spectrum was deconvoluted into two peaks centered at 398.2 eV and 399.0 eV, corresponding to N–B and N–H bonds, respectively, with the N–H portion estimated to be 2.9%.


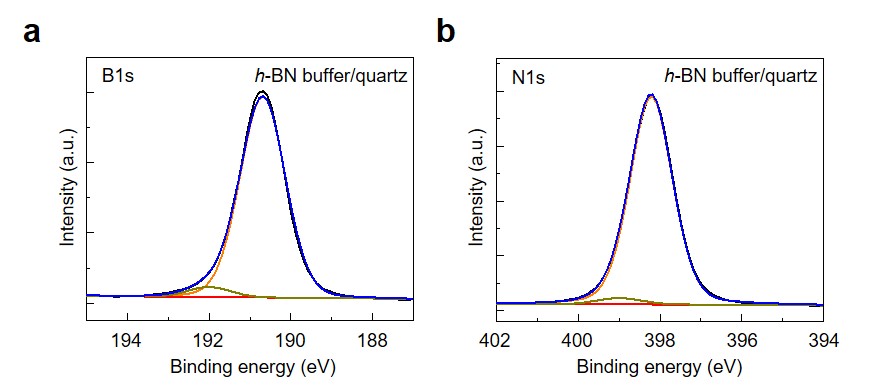


**Figure S7.** High-resolution X-ray photoelectron spectroscopy (XPS) spectra of a) B1s and b) N1s for the *h*-BN buffer/quartz sample.

**Supplementary Note 5. Two Growth Processes for Stable HBN**

For the growth of *h*-BN buffer, a quartz substrate (NC-200, Jinzhou new century quartz glass) was placed into a 2” quartz tube CVD system, as illustrated in Figure S5. The temperature was ramped up to 900°C within 15 min under a flow of H_2_ (1,000 sccm) at a pressure of 3.4 torr. At 900°C, the *h*-BN buffer was grown for 20 min using N_2_ (1,000 sccm) as a carrier gas for B_3_N_3_H_6_ with O_2_ (0.2 sccm) introduced simultaneously. Growth was then continued while ramping the temperature up to 1,050°C for 30 min at a pressure of 3.8 torr to form a quality gradient. As shown in Figure S6, the resulting *h*-BN buffer thickness was ~57 nm.

Two different processes were tested for the subsequent stable HBN growth:

1. Continuous Process

- HBN growth proceeds immediately after the *h*-BN buffer growth without quenching to room temperature.

1. Discontinuous Process

- HBN growth includes an intermediate quenching step to room temperature after the *h*-BN buffer growth.

In both processes, HBN was grown at 1,050°C by increasing the N_2_ flow rate from 100 to 1,000 sccm over the desired growth duration. The flows of O_2_ and CH_4_ were also adjusted to achieve the target HBN composition.


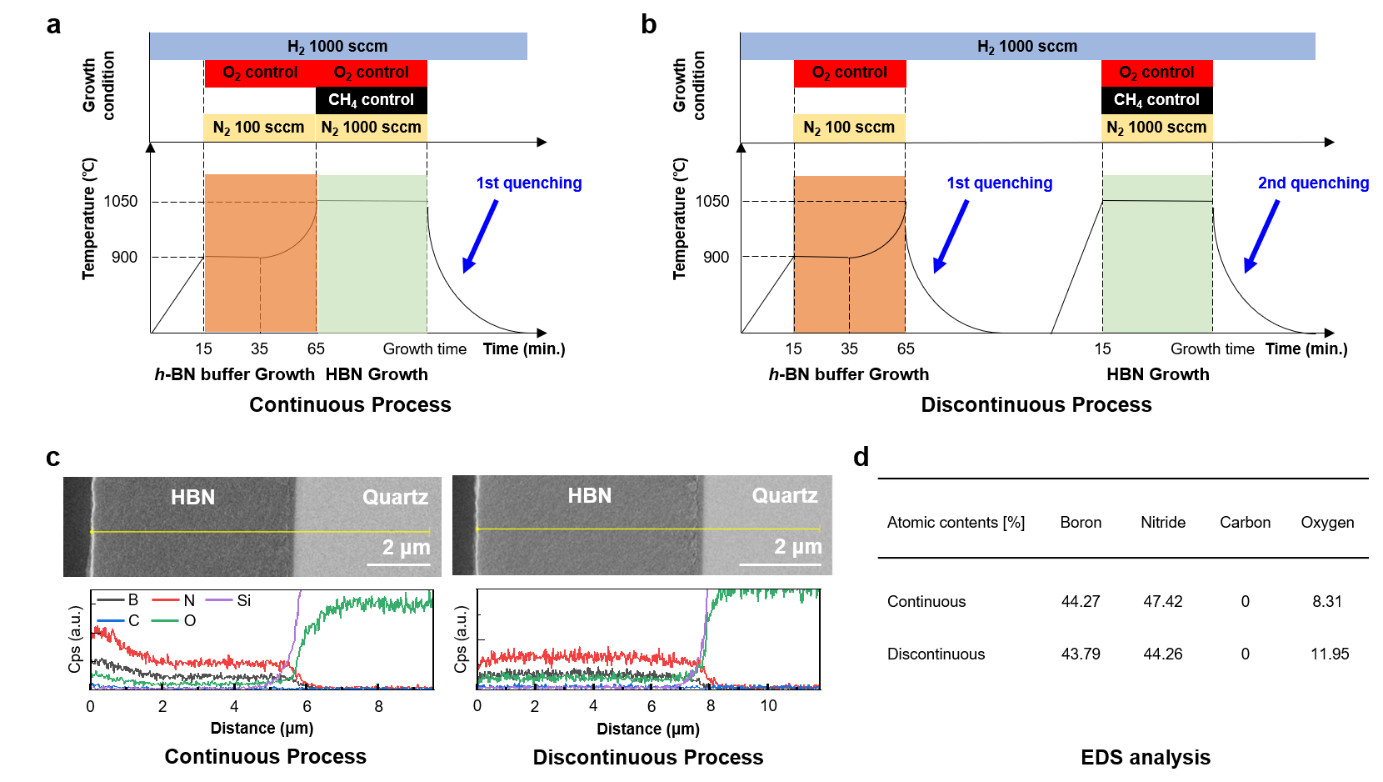


**Figure S8.** HBN growth profiles of a) continuous process and b) discontinuous process.

c) Cross-sectional scanning electron microscopy (SEM) images and corresponding energy dispersive spectrometer (EDS) line scanning profiles of HBNs grown by the continuous and the discontinuous processes, respectively. d) Summary of the atomic compositions of HBNs from each process.


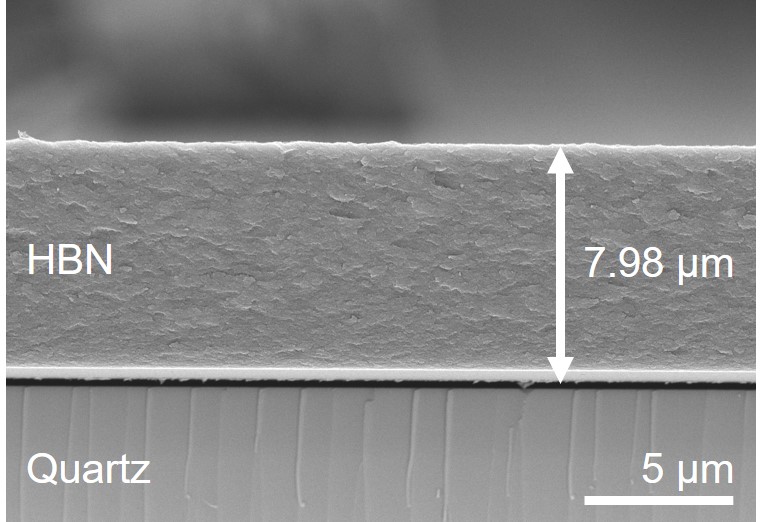


**Figure S9.** Cross-sectional SEM image showing the spontaneous delamination of the HBN film from the quartz growth substrate~~,~~ over time.

**Supplementary Note 6. Density Estimation of HBN by Neutron****-Shielding Probability**

Figure S10a,b shows the 25 meV thermal-neutron capturing probability as a function of thickness of *h*-BN, *P*(*T_h_*_-BN_) from the Ref [2], and for HBN, *P*(*T*_HBN_), obtained from our measurements. The capturing probability of *h*-BN can be expressed as a Ref [2]:

$P\left( T_{h-BN} \right)=1-\exp\left( -\frac{T_{h-BN}}{\lambda_{h-BN}} \right).$ (1)

where *λ_h_*_-BN_ is the thermal-neutron absorption length of *h*-BN (237 μm for natural *h*-BN) and *T_h_*_-BN_ is the thickness of *h*-BN.

Similarly, the capturing probability of HBN can be expressed as:

$P\left( T_{H\mathrm{BN}} \right)=1-exp(-\frac{T_{\mathrm{HBN}}}{\lambda_{\mathrm{HBN}}})$. (2)

where *T*_HBN_ is the HBN thickness, and *λ*_HBN_ is 230.1 μm. The value of *λ*_HBN_ was derived by scaling *λ_h_*_-BN_ with the thickness ratio of *h*-BN and HBN at equivalent neutron-shielding efficiency. Specifically, the shielding efficiency was 16.1% at 41.6 μm for *h*-BN and at 40.4 μm for HBN, yielding a ratio of 0.97 (Figure S10).

Since the thermal-neutron cross-section of ^10^B ^[3]^ is more than 2,000 times higher than that of N, C, and O ^[4, 5]^, the neutron-shielding efficiency of HBN is primarily governed by the B atomic density. At a fixed thickness, this density is determined by both the B atomic content and the bulk density of HBN. The equivalent thickness of HBN that provides the same shielding efficiency as *h*-BN can therefore be expressed as:

$T_{HBN}=\alpha{\times\beta\times T}_{h-BN}.$ (3)

where *α* is the ratio of the B atomic content ratios of *h*-BN to that of HBN, and *β* is the density to that of *h*-BN. From the experimentally observed thickness ratio of *h*-BN to HBN at the same shielding efficiency of 0.97 (*α* × *β*), it follows that HBN achieves the same neutron-shielding probability as *h*-BN with a 3% thinner film. Given that the B content of HBN is 0.39, *α* is calculated to be 1.282. Consequently, *β* is determined as 0.757. Since *β* is the ratio of the density of HBN to that of *h*-BN (2.28 g cm^-3^) ^[6]^, the density of HBN is evaluated to be 3.01 g cm^-3^.


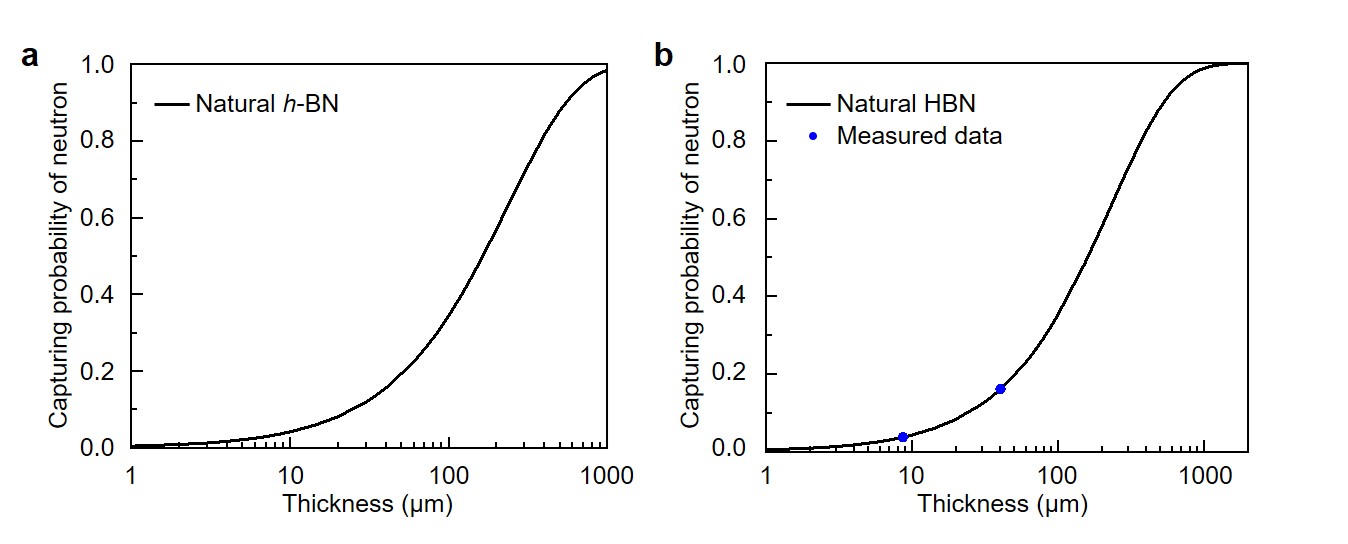


**Figure S10.** The probability of thermal neutron interaction for a) *h*-BN ^[2]^ and b) HBN.


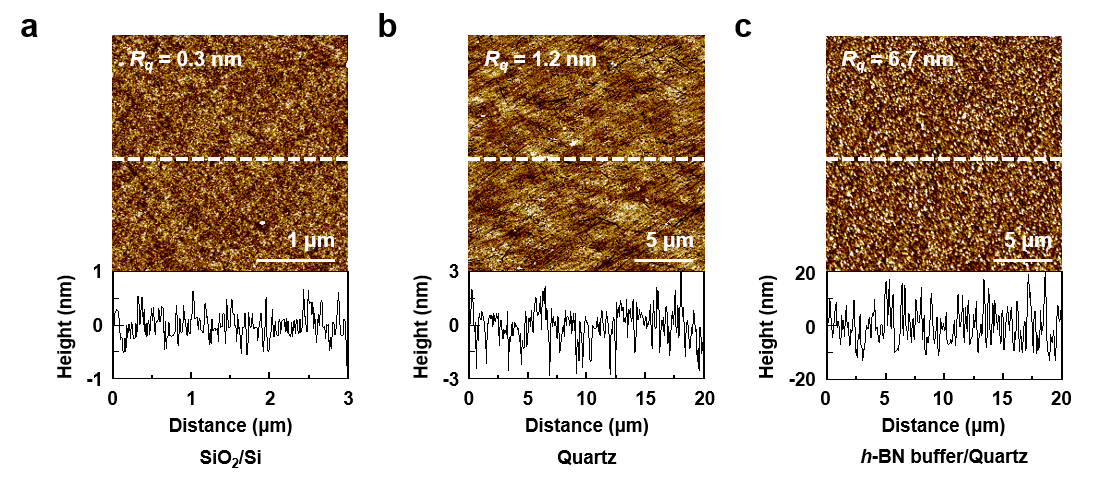


**Figure S11.** AFM analysis of surface topographies, line profiles, and root-mean-square roughness (*R_q_*) for a) SiO_2_/Si wafer, b) bare quartz, and c) *h*-BN buffer/quartz.


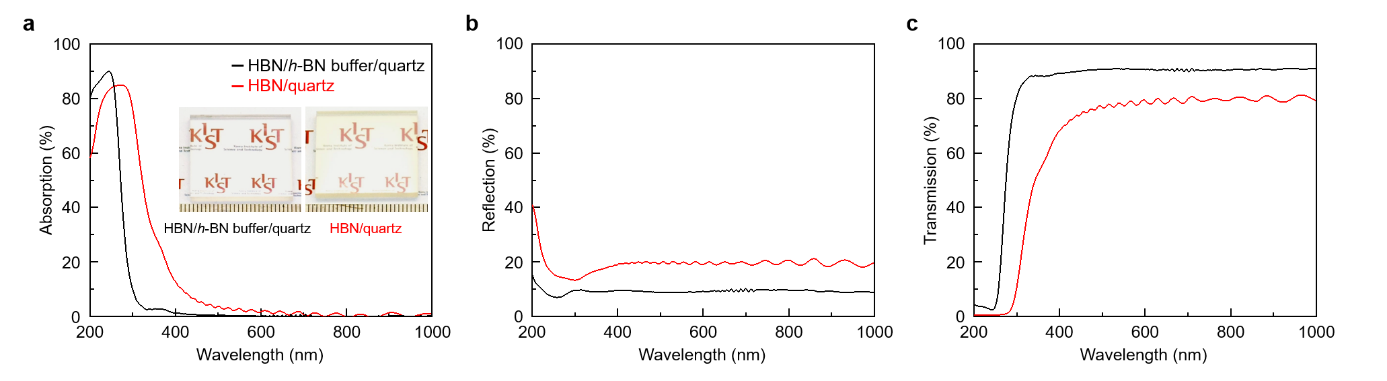


**Figure S12.** Ultraviolet-visible spectroscopy (UV-Vis) characterization of HBN films on different substrates: a) absorption, b) reflection, and c) transmission spectra of HBN/*h*-BN buffer/quartz (black) and HBN/quartz (red). Insets show photographs of the corresponding HBN samples.


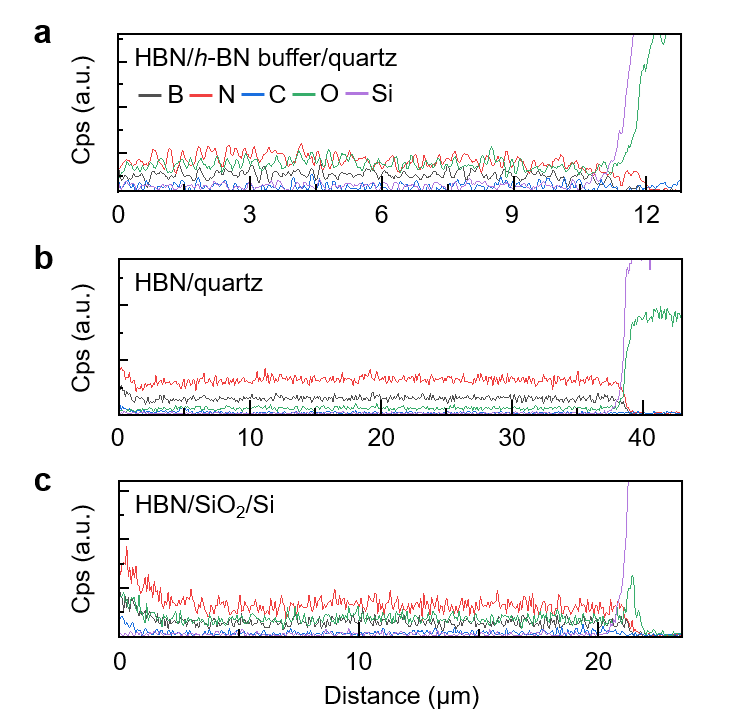


**Figure S13.** EDS raw line scanning profiles of HBN films grown on a) *h*-BN buffer/quartz, b) quartz, and c) SiO_2_/Si, respectively.


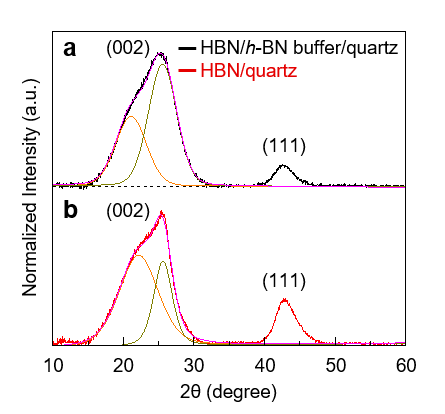


**Figure S14.** Deconvoluted X-ray diffraction (XRD) spectra of HBN films grown on a) *h*-BN buffer/quartz (black) and b) quartz (red) substrates.

**Supplementary Note 7. PL Measurement Setup for Optically Optimized HBN**

Photoluminescence (PL) measurements of optically optimized HBN were performed using a continuous wave (CW) 532 nm laser (MGL-III-532, CNI Laser) for excitation. The laser power at each excitation frequency was measured before the objective lens using a PM100USB (Thorlabs). Emission spectra were recorded with a CCD camera (DU401A-BVF, Andor) mounted on a spectrometer (MonoRa 512i, Dongwoo Optron, Oxford Instruments). After passing through a beam splitter (BS) and a 550 nm long-pass filter (LPF), the PL intensity was detected using an avalanche photodiode (APD) module (SPCM-AQRH, Excelitas).


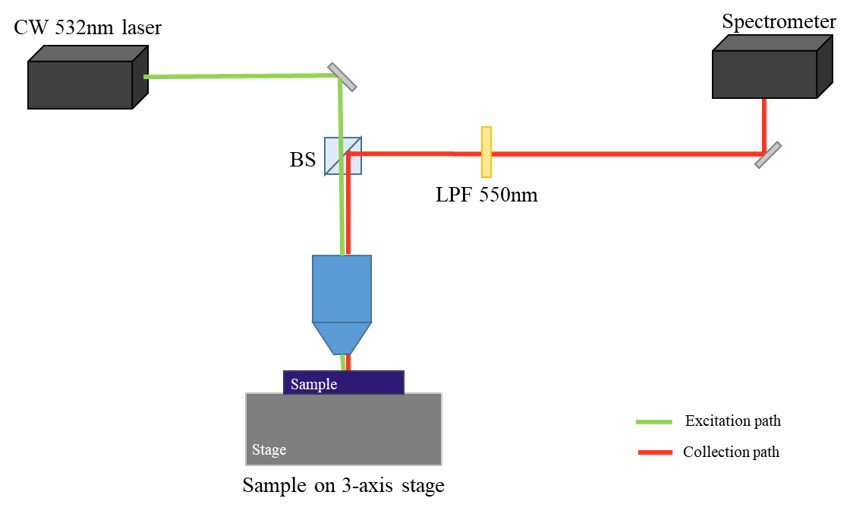


**Figure S15.** Schematic of the photoluminescence (PL) measurement setup.


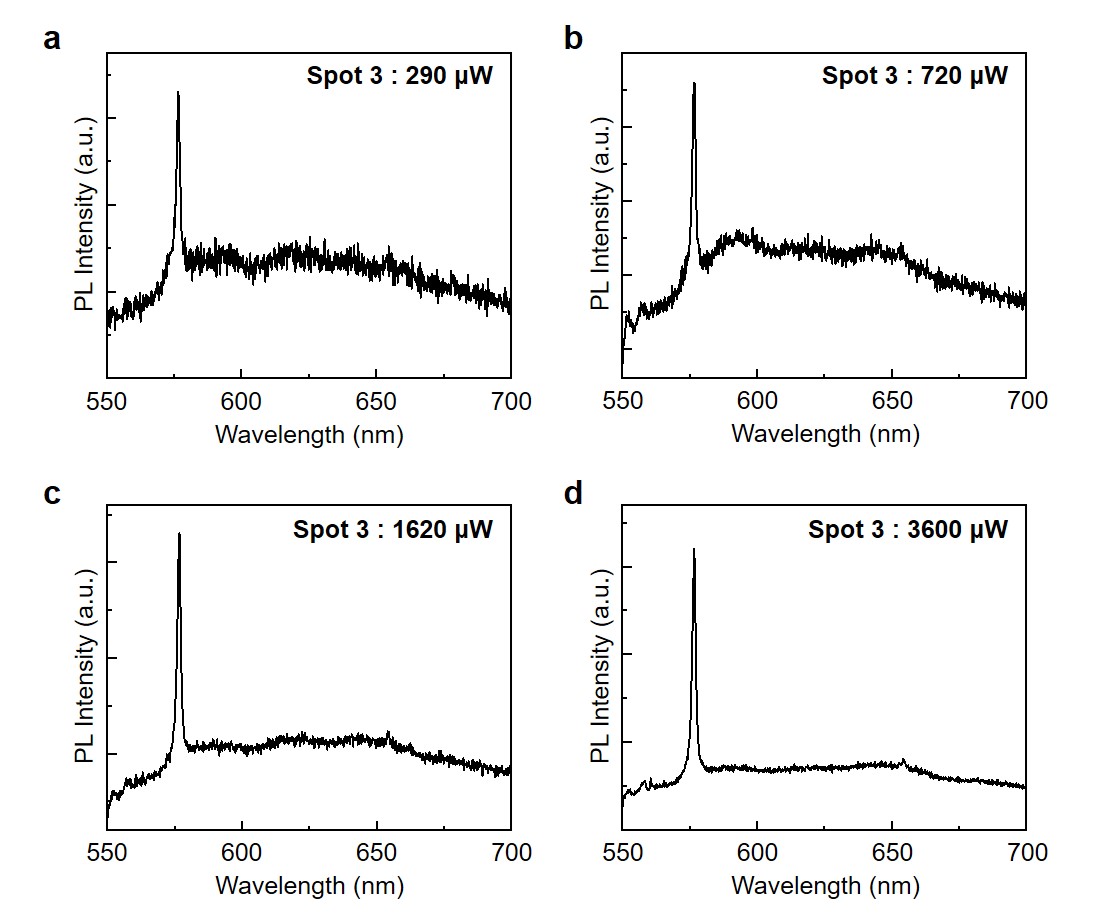


**Figure S16.** PL spectra measured at spot 3 for laser powers of a) 290 μW, b) 720 μW, c) 1620 μW, and d) 3600 μW.


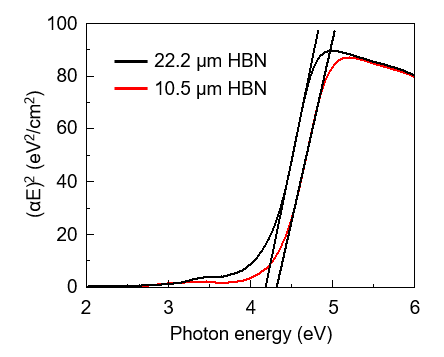


**Figure S17.** UV-Vis absorption spectra of HBN films with varying thicknesses, used for the determination of the optical band-gap.


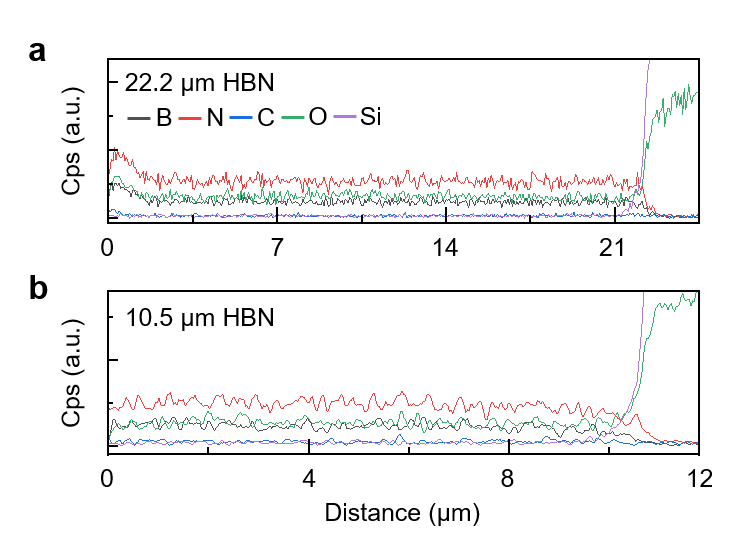


**Figure S18.** EDS raw line scanning profiles of a) 22.2 μm and b) 10.5 μm HBN films grown on *h*-BN buffer/quartz.

**Supplementary Note 8. Estimation of sp^3^ Fraction and Density of HBN from XRD**

The sp^3^ fraction (*P*_sp3_) of HBN was estimated using deconvoluted XRD peaks of the overall sp^2^ (002) and sp^3^ (111) reflections (Figure S19). Assuming an ideal B_0.5_N_0.5_ composition, the bulk density of HBN (*ρ*_HBN_) is expressed as:

$\rho_{\mathrm{HBN}}= \rho_{overall sp2}\times\left( 100-P_{sp3} \right)+ \rho_{sp3}\times P_{sp3}.$ (4)

where *ρ*_overall sp2_ and *ρ*_sp3_ are the densities of overall sp^2^ (002) and sp^3^ (111) components. The overall sp^2^ density is further decomposed to include contributions from pure sp^2^ and sp^2^-sp^3^ hybridized structures:

$\rho_{overall sp2}= \rho_{sp2}\times\left( 100-P_{sp2-sp3} \right)+ \rho_{sp2-sp3}\times P_{sp2-sp3}.$ (5)

where *P*_sp2-sp3_ is 23.7%, which is the fraction of the sp^2^-sp^3^ (002) peak area in the overall sp^2^ (002) peak area. Since density is inversely proportional to the lattice spacing, the corresponding values can be calculated as:

$\rho_{sp2}= \rho_{h-BN}\times\frac{d_{h-BN}}{d_{sp2}}.$ (6)

$\rho_{sp2-sp3}= \rho_{h-BN}\times\frac{d_{h-BN}}{d_{sp2-sp3}}.$ (7)

where *d_h_*_-BN_, *d*_sp2_​, and *d*_sp2-sp3_​ are the measured d-spacings of *h*-BN (0.333 nm) ^[6]^, sp^2^ (002) (0.348 nm), and sp^2^-sp^3^ (002) (0.421 nm) ^[7]^, respectively. From these values, *ρ*_sp2_ and *ρ*_sp2-sp3_ were calculated as 2.18 and 1.80 g cm^-3^. The overall sp^2^ density is then *ρ*_overall sp2_ = 2.09 g cm^-3^.

Considering the three-dimensional cubic structure of *c*-BN, the density of the sp^3^ phase (*ρ*_sp3_) was calculated based on the known *c*-BN density (*ρ_c_*_-BN_) and the lattice expansion of the sp^3^ (111) planes:

$\rho_{sp3}= \rho_{c-BN}\times{(\frac{a_{c-BN}}{a_{sp3}})}^{3}.$ (7)

where *a_c_*_-BN_ and *a*_sp3_ are the lattice constants of *c*-BN (0.362 nm) ^[6]^ and sp^3^ (111) (0.367 nm). Using *ρ_c_*_-BN_ (3.49 g cm^-3^), *ρ*_sp3_ was calculated to be 3.35 g cm^-3^.

To account for the actual measured composition (B_0.42_N_0.42_C_0.01_O_0.15_), the density was corrected:

${\rho'}_{\mathrm{HBN}}= {[\rho}_{overall sp2}\times\left( 100-P_{sp3} \right)+ \rho_{sp3}\times P_{sp3}] \times\frac{W_{\mathrm{HBN}}}{W_{h-BN}}.$ (8)

where *W*_HBN_ and *W_h_*_-BN_ are the atomic weights of HBN (1293.6) and *h*-BN (1240). Using *ρ´*_HBN_ = 3.01 g cm^-3^ from Figure S10 and Equation (8), *P*_sp3_ is calculated to be 63.4%.

From the XRD area, the sp^3^ portion in HBN (*P´*_sp3_) can be expressed as follows:

${P'}_{sp3}= \frac{1}{1+A_{overall sp2}}\times100$ (9)

where *A*_overall sp2_ is the overall sp^2^ (002) peak area normalized by the sp^3^ (111) peak area, which is 8.48. The *P´*_sp3_ calculated using Equation (9) is 10.6%, while the actual *P*_sp3_ is 63.4%, requiring a correction that can be expressed as follows:

$P_{sp3}= \frac{C}{C+A_{overall sp2}}\times100$ (10)

where *C* is the correction factor for sp^3^ (111) peak area. Using *P*_sp3_ (63.4%) and Equation (10), the *C* is calculated to be 14.69_._


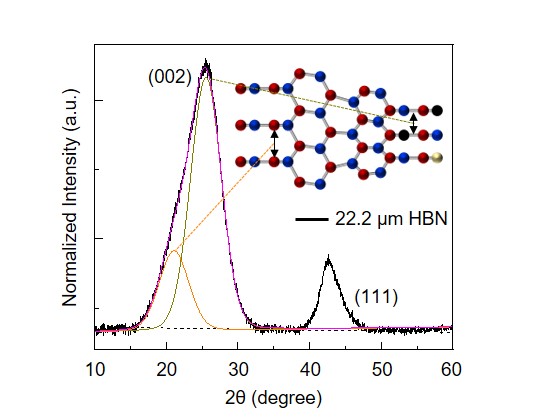


**Figure S19.** Deconvolution of the XRD spectrum of 22.2 μm HBN: inset shows the corresponding HBN structures for each deconvoluted (002) peak.


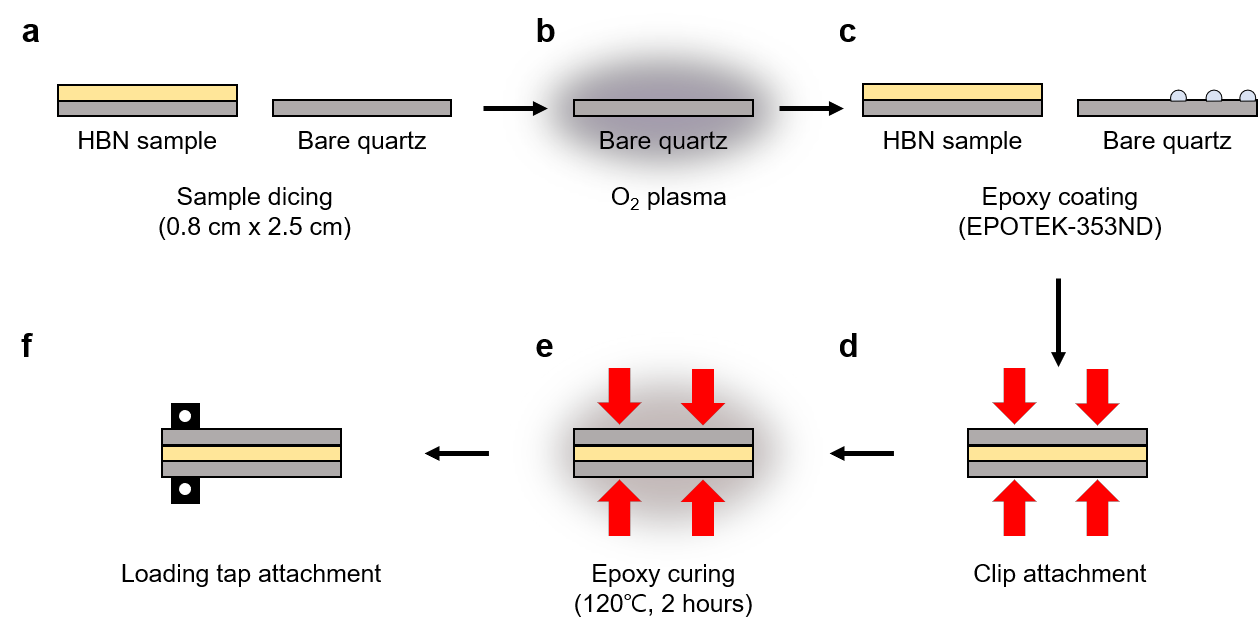


**Figure S20.** Fabrication procedure of the double cantilever beam (DCB) specimen. a) Dicing of the HBN/*h*-BN buffer/quartz sample and a counterpart quartz substrate to the same size. b) O_2_ plasma treatment on bare quartz. c) Epoxy coating on the sample. d) Attaching the two substrates under constant clamping pressure. e) Epoxy curing at 120°C for 2 h under clamping pressure. f) Attachment of the loading tap.


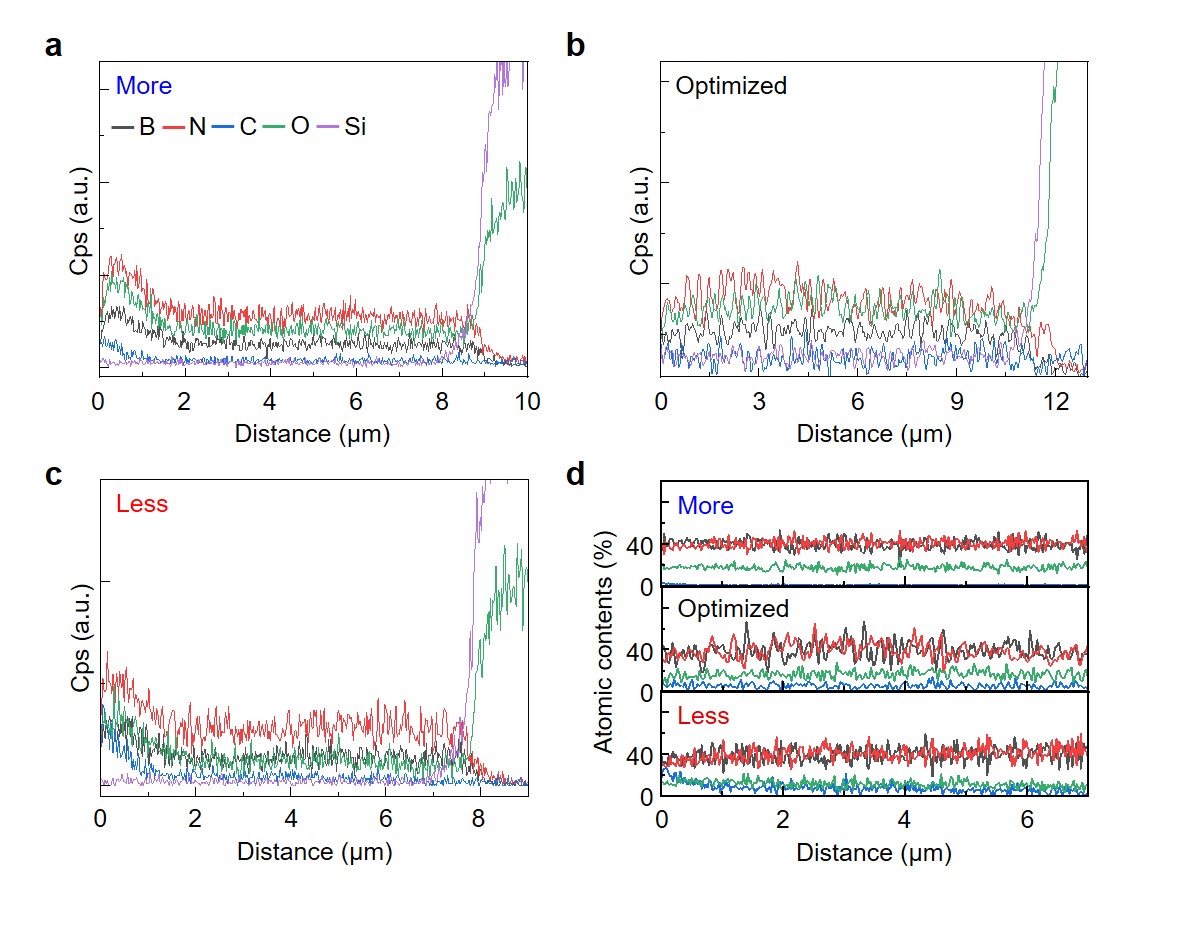


**Figure S21.** EDS raw line scanning profiles of ~10 μm HBN samples. a) High O contents (More), b) Optimized O contents (Optimized), and c) Low O contents (Less), each compared to optically optimized HBN. d) Summary of elemental profiles of More, Optimized, and Less HBNs.

References

[1] A. Das, D. J. Lee, P. K. Shandilya, S. Kim, G. Kang, D. P. Lake, B. Behera, D. Sukachev, I. Aharonovich, J.-H. Lee, J. Park, P. E. Barclay, *ACS Photonics* **2021**, *8*, 3027.

[2] T. C. Doan, J. Li, J. Y. Lin, H. X. Jiang, *AIP Adv.* **2016**, *6*, 075213.

[3] J. Meadows, J. Whalen, *Nucl. Sci. Eng.* **1970**, *40*, 12.

[4] R. Adeli, S. P. Shirmardi, H. Abbasi, S. J. Ahmadi, *Sci. Eng. Compos. Mater.* **2018**, *25*, 725.

[5] R. B. Firestone, Zs. Revay, *Phys. Rev. C* **2016**, *93*.

[6] M. Z. Karim, D. C. Cameron, M. S. J. Hashmi, *Surf. Coat. Technol.* **1993**, *60*, 502.

[7] S. Komatsu, *J.Phys. D: Appl. Phys.* **2007**, *40*, 2320.
